# Identification of Novel Genetic Markers Associated with Clinical Phenotypes of Systemic Sclerosis through a Genome-Wide Association Strategy

**DOI:** 10.1371/journal.pgen.1002178

**Published:** 2011-07-14

**Authors:** Olga Gorlova, Jose-Ezequiel Martin, Blanca Rueda, Bobby P. C. Koeleman, Jun Ying, Maria Teruel, Lina-Marcela Diaz-Gallo, Jasper C. Broen, Madelon C. Vonk, Carmen P. Simeon, Behrooz Z. Alizadeh, Marieke J. H. Coenen, Alexandre E. Voskuyl, Annemie J. Schuerwegh, Piet L. C. M. van Riel, Marie Vanthuyne, Ruben van 't Slot, Annet Italiaander, Roel A. Ophoff, Nicolas Hunzelmann, Vicente Fonollosa, Norberto Ortego-Centeno, Miguel A. González-Gay, Francisco J. García-Hernández, María F. González-Escribano, Paolo Airo, Jacob van Laar, Jane Worthington, Roger Hesselstrand, Vanessa Smith, Filip de Keyser, Fredric Houssiau, Meng May Chee, Rajan Madhok, Paul G. Shiels, Rene Westhovens, Alexander Kreuter, Elfride de Baere, Torsten Witte, Leonid Padyukov, Annika Nordin, Raffaella Scorza, Claudio Lunardi, Benedicte A. Lie, Anna-Maria Hoffmann-Vold, Øyvind Palm, Paloma García de la Peña, Patricia Carreira, Spanish Scleroderma Group, John Varga, Monique Hinchcliff, Annette T. Lee, Pravitt Gourh, Christopher I. Amos, Frederick M. Wigley, Laura K. Hummers, J. Hummers, J. Lee Nelson, Gabriella Riemekasten, Ariane Herrick, Lorenzo Beretta, Carmen Fonseca, Christopher P. Denton, Peter K. Gregersen, Sandeep Agarwal, Shervin Assassi, Filemon K. Tan, Frank C. Arnett, Timothy R. D. J. Radstake, Maureen D. Mayes, Javier Martin

**Affiliations:** 1 Department of Epidemiology, M. D. Anderson Cancer Center, Houston, Texas, United States of America; 2 Instituto de Parasitología y Biomedicina López-Neyra, Consejo Superior de Investigaciones Científicas, Granada, Spain; 3 Department of Medical Genetics, University Medical Center Utrecht, Utrecht, The Netherlands; 4 Department of Rheumatology, Radboud University Nijmegen Medical Center, Nijmegen, The Netherlands; 5 Servicio de Medicina Interna, Hospital Valle de Hebron, Barcelona, Spain; 6 University Medical Centre Groningen, Department of Epidemiology, Groningen, The Netherlands; 7 Department of Human Genetics, Radboud University Nijmegen Medical Center, Nijmegen, The Netherlands; 8 VU University Medical Center, Amsterdam, The Netherlands; 9 Department of Rheumatology, University of Leiden, Leiden, The Netherlands; 10 Cliniques Universitaires Saint-Luc, Université Catholique de Louvain, Brussels, Belgium; 11 Department of Dermatology, University of Cologne, Cologne, Germany; 12 Servicio de Medicina Interna, Hospital Clínico Universitario, Granada, Spain; 13 Servicio de Reumatología, Hospital Marqués de Valdecilla, Santander, Spain; 14 Servicio de Medicina Interna, Hospital Virgen del Rocio, Sevilla, Spain; 15 Servicio de Inmunología, Hospital Virgen del Rocío, Sevilla, Spain; 16 Rheumatology Unit and Chair, Spedali Civili, Università degli Studi, Brescia, Italy; 17 Institute of Cellular Medicine, Newcastle University, Newcastle Upon Tyne, United Kingdom; 18 Department of Rheumatology and Epidemiology, University of Manchester, Manchester Academic Health Science Centre, Manchester, United Kingdom; 19 Department of Clinical Sciences, Division of Rheumatology, Lund University, Lund, Sweden; 20 Ghent University, Ghent, Belgium; 21 Centre for Rheumatic Diseases, Glasgow Royal Infirmary Glasgow, United Kingdom; 22 Department of Surgery, Western Infirmary Glasgow, University of Glasgow, Glasgow, United Kingdom; 23 Katholieke Universiteit Leuven, Leuven, Belgium; 24 Department of Dermatology, Josefs-Hospital, Ruhr University Bochum, Germany; 25 Center for Medical Genetics, Ghent University Hospital, Ghent, Belgium; 26 Hannover Medical School, Hannover, Germany; 27 Center for Molecular Medicine, Karolinska Institutet, Stockholm, Sweden; 28 Referral Center for Systemic Autoimmune Diseases, Fondazione IRCCS Ca' Granda Ospedale Maggiore Policlinico and University of Milan, Milan, Italy; 29 Department of Medicine, Policlinico GB Rossi, University of Verona, Italy; 30 Institute of Immunology, Oslo University Hospital Rikshospitalet, Oslo, Norway; 31 Department of Rheumatology, Rikshospitalet, Oslo University Hospital, Oslo, Norway; 32 Servicio de Reumatología, Hospital Ramón y Cajal, Madrid, Spain; 33 Hospital 12 de Octubre, Madrid, Spain; 34 Northwestern University Feinberg School of Medicine, Chicago, Illinois, United States of America; 35 Feinstein Institute of Medical Research, Manhasset, New York, United States of America; 36 The University of Texas Health Science Center–Houston, Houston, Texas, United States of America; 37 The Johns Hopkins University Medical Center, Baltimore, Maryland, United States of America; 38 Fred Hutchinson Cancer Research Center, Seattle, Washington, United States of America; 39 Department of Rheumatology and Clinical Immunology, Charité University Hospital, Berlin, Germany; 40 Centre for Rheumatology, Royal Free and University College School, London, United Kingdom; ¤ For membership of the Spanish Scleroderma Group, please see Text S1.; University of Oxford, United Kingdom

## Abstract

The aim of this study was to determine, through a genome-wide association study (GWAS), the genetic components contributing to different clinical sub-phenotypes of systemic sclerosis (SSc). We considered limited (lcSSc) and diffuse (dcSSc) cutaneous involvement, and the relationships with presence of the SSc-specific auto-antibodies, anti-centromere (ACA), and anti-topoisomerase I (ATA). Four GWAS cohorts, comprising 2,296 SSc patients and 5,171 healthy controls, were meta-analyzed looking for associations in the selected subgroups. Eighteen polymorphisms were further tested in nine independent cohorts comprising an additional 3,175 SSc patients and 4,971 controls. Conditional analysis for associated SNPs in the HLA region was performed to explore their independent association in antibody subgroups. Overall analysis showed that non-HLA polymorphism rs11642873 in *IRF8* gene to be associated at GWAS level with lcSSc (*P* = 2.32×10^−12^, OR = 0.75). Also, rs12540874 in *GRB10* gene (*P* = 1.27 × 10^−6^, OR = 1.15) and rs11047102 in *SOX5* gene (*P* = 1.39×10^−7^, OR = 1.36) showed a suggestive association with lcSSc and ACA subgroups respectively. In the HLA region, we observed highly associated allelic combinations in the *HLA-DQB1* locus with ACA (*P* = 1.79×10^−61^, OR = 2.48), in the *HLA-DPA1/B1* loci with ATA (*P* = 4.57×10^−76^, OR = 8.84), and in *NOTCH4* with ACA *P* = 8.84×10^−21^, OR = 0.55) and ATA (*P* = 1.14×10^−8^, OR = 0.54). We have identified three new non-HLA genes (*IRF8, GRB10,* and *SOX5*) associated with SSc clinical and auto-antibody subgroups. Within the HLA region, *HLA-DQB1, HLA-DPA1/B1*, and *NOTCH4* associations with SSc are likely confined to specific auto-antibodies. These data emphasize the differential genetic components of subphenotypes of SSc.

## Author Summary

Scleroderma or systemic sclerosis is a complex autoimmune disease affecting one individual of every 100,000 in Caucasian populations. Even though current genetic studies have led to better understanding of the pathogenesis of the disease, much remains unknown. Scleroderma is a heterogeneous disease, which can be subdivided according to different criteria, such as the involvement of organs and the presence of specific autoantibodies. Such subgroups present more homogeneous genetic groups, and some genetic associations with these manifestations have already been described. Through reanalysis of a genome-wide association study data, we identify three novel genes containing genetic variations which predispose to subphenotypes of the disease (*IRF8, GRB10,* and *SOX5*). Also, we better characterize the patterns of associated loci found in the HLA region. Together, our findings lead to a better understanding of the genetic component of scleroderma.

## Introduction

Genetic factors play an essential role in scleroderma or systemic sclerosis (SSc) etiology as in most complex autoimmune diseases [1]. Multiple reports of well powered candidate gene association and replication studies, together with the first genome-wide association study (GWAS) in this disease have led to the establishment of the Major histocompatibility complex (MHC), *STAT4, IRF5, BLK, BANK1, TNFSF4* and *CD247* as SSc susceptibility genes [2]–[15].

SSc is a clinically heterogeneous disease with a wide range of clinical manifestations, ranging from mild skin fibrosis with minimal internal organ disease to severe skin and organ involvement, reflecting the three main pathological events that characterize this disease: endothelial damage, fibrosis, and autoimmune dysregulation [16]. SSc patients are classified into two clinical subgroups based on the extent of skin involvement, limited SSc (lcSSc) and diffuse SSc (dcSSc) that are associated with different clinical complications and prognoses [17]. Another SSc hallmark is the presence of disease specific and usually mutually exclusive auto-antibodies that correlate both with the extent of skin involvement and the various disease manifestations, such as pulmonary fibrosis and renal crisis [18]. The most common are DNA topoisomerase I (ATA), and anti-centromere antibodies (CENP A and/or B proteins) [19]. Each of these auto-antibodies is a marker for relatively distinct clinical subgroups of SSc, with anti-centromere typically associated with limited cutaneous disease, uncommon pulmonary fibrosis, late-onset pulmonary hypertension but generally an overall good prognosis, while ATA is a marker for diffuse skin disease and clinically significant pulmonary fibrosis with a resultant poorer prognosis.

It has been observed that certain SSc clinical features and the presence of disease specific auto-antibodies vary in different countries and ethnicities [20]. This fact supports the likelihood that genetic factors may influence the different clinical features of the disease and auto-antibody subsets [19]. Furthermore, the affected members within multicase SSc families tend to be concordant for SSc-specific auto-antibodies and HLA haplotypes, thus, providing further evidence for a genetic basis for auto-antibody expression in SSc [21]. Moreover, several studies have reported that certain SSc genetic risk factors correlate with specific clinical subsets of the disease or SSc-related auto-antibodies [4], .

In this study, we aimed to identify novel genetic factors associated with different SSc clinical and auto-antibody subsets through a stratified re-analysis of results from a previous GWAS from our group and validation in a large replication study.

## Results

First, the genetic associations were tested in each of the four subgroups considered for this study (lcSSc, dcSSc, ACA positive and ATA positive) by the means of *χ2* tests in the GWAS data (individuals from the United States, Spain, Germany and The Netherlands), correcting the *P* values for the genomic inflation factor λ of each subgroup (Figures S1, S2, S3, S4 and Tables S1, S2, S3, S4). We found a total of eighteen novel non-HLA loci associated in these subgroups with a *P* value lower than 1×10^−5^, seven in the lcSSc subtype, five in the dcSSc subtype, two in ACA positives and four in ATA positives. Next, we proceeded to replicate these associations in nine independent cohorts (from US, Spain, Germany, The Netherlands, Belgium, Italy, Sweden, United Kingdom and Norway). The statistically significant results observed in the replication step are shown in Table 1. The complete set of data is shown in Tables S1, S2, S3 S4.

**Table 1:** Novel non-HLA loci associated with SSc clinical and serological subtypes.

SSc Subphenotype	Chr.	Gene	SNP	Base Pair	Location	Change	Stage	N (case/control)	MAF (case/control)	*P* † value	OR (95% CI)
lcSSc	7p12.1	*GRB10*	rs12540874	50,632,416	Intronic	G/A	GWAS	1400/5172	0.461/0.409	3.00×10^−6^	1.23 (1.13-1.34)
							Replication	1960/4971	0.416/0.395	3.07×10^−2^	1.09 (1.01–1.18)
							Combined	3360/10143	0.435/0.403	1.27×10^−6^	1.15 (1.09–1.22)
	16q24.1	*IRF8*	rs11642873	84,549,206	Intergenic	C/A	GWAS	1400/5172	0.144/0.197	1.39×10^−7^	0.72 (0.64–0.81)
							Replication	1960/4971	0.143/0.186	6.88×10^−6^	0.78 (0.70–0.87)
							Combined	3360/10143	0.144/0.192	2.32×10^−12^	0.75 (0.69–0.81)
dcSSc	12q13.2	*RPL41/ESYT1* *	rs11171747	54,804,675	Upstream	G/T	GWAS	740/5172	0.446/0.384	2.19×10^−6^	1.31 (1.01–1.29)
							Replication	959/4971	0.408/0.372	3.49×10^−3^	1.16 (1.15–1.71)
							Combined	1699/10143	0.425/0.379	5.99×10^−8^	1.23 (1.10–1.50)
ACA+	12p12.1	*SOX5*	rs11047102	23,837,413	Intronic	T/C	GWAS	761/5172	0.132/0.096	1.03×10^−5^	1.47 (1.24–1.73)
							Replication	1030/4971	0.123/0.102	2.91×10^−3^	1.27 (1.09–1.48)
							Combined	1791/10143	0.127/0.099	1.39×10^−7^	1.36 (1.21–1.52)

**†:** *P* values for GWAS cohorts are Mantel-Haenszel meta-analysis GC corrected according to the set λ and in the replication and combined analysis Mantel-Haenszel meta-analysis *P* value.

*Association in rs11171747 had a significant BD *P* value, thus making it heterogeneous association among populations.

In addition, exhaustive analysis was performed in the HLA region (megabases 28 to 34 in chromosome 6) with the GWAS data in order to find specific subgroup associations in this region. Due to the fact that most associations found herein in the MHC region have been previously described, we did not perform a replication phase of these findings. Instead, let these results be the replication for previous works. It is also noteworthy that all independent associations found within the MHC region have almost exactly the same ORs in the four GWAS cohorts separately, thus, replicating themselves.

### Clinical Manifestations

**Figure 1:**
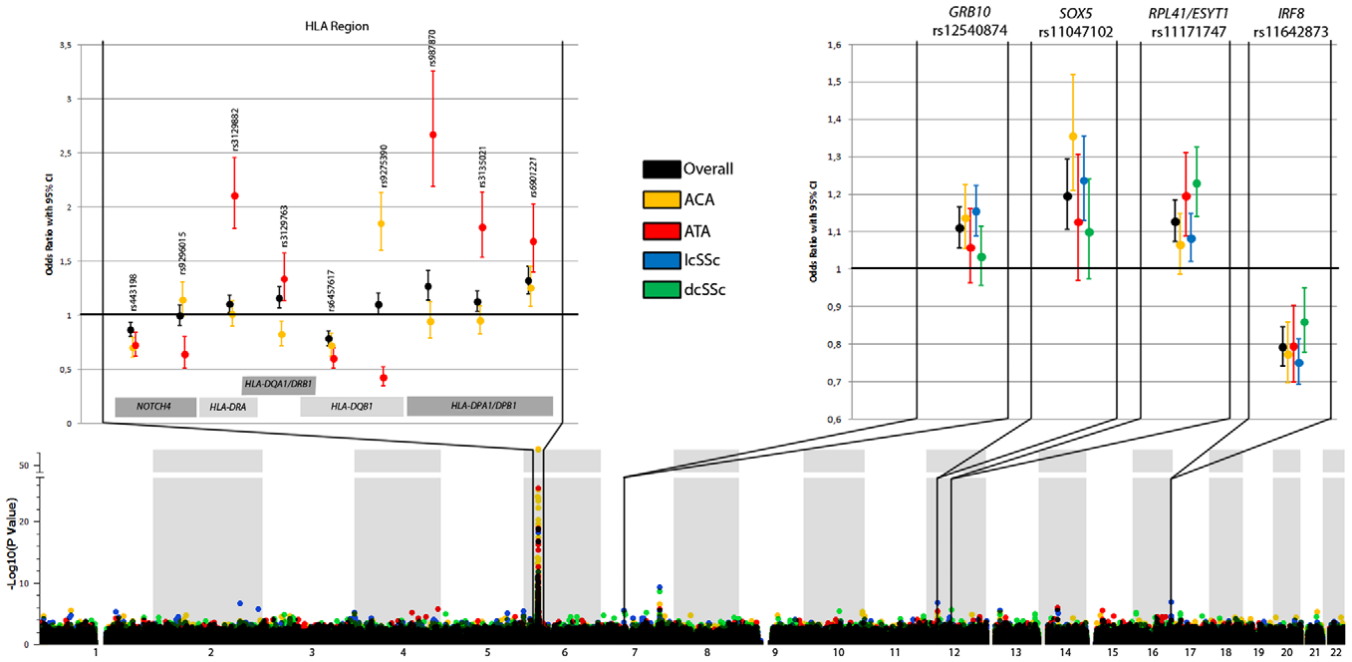
New loci associated with subphenotypes of SSc. The lower part shows the Manhattan Plot with corrected *P* values of the GWAS cohorts. The upper part shows the ORs and the 95% CI interval of the novel associated regions in the GWAS cohorts (HLA region, left panel) and all cohorts (non-HLA loci, right panel) for the overall analysis and each subphenotype considered in the study. (Note: the ORs and CIs on the forest plot do not exactly correspond to the numbers in Table 1 and Table 2. Table 1 and Table 2 shows marginal effects of these SNPs while this figure presents ORs and CIs after the adjustment for the other SNPs claimed as independent for that phenotype).

In the lcSSc subtype, seven non-HLA novel loci were identified as susceptibility markers in the GWAS data (Table S1 and Figure S1). Two out of the seven genetic markers showed evidence of association in the replication cohorts: rs11642873 near the *IRF8* gene (lcSSc *P* = 2.32×10^−12^, OR = 0.75 [0.69–0.81]) at the GWAS level of significance and rs12540874 in the *GRB10* gene (lcSSc *P* = 1.27×10^−6^, OR = 1.15 [1.09–1.22]) at the suggestive level of significance (Figure 1, Table 1 and Table S1).

**Table 2:** Independent associations identified in the HLA region with the ACA and ATA positive subgroups.

						ACA				ATA			
						Unadjusted		Adjusted		Unadjusted		Adjusted	
SSc Subphenotype	SNP	Gene	Location	Change	MAF (ACA/ATA/control)	*P* †	OR (CI 95%)	*P* *	OR (CI 95%)	*P* †	OR (CI 95%)	*P* *	OR (CI 95%)
ACA+	rs443198	*NOTCH4*	Exon	C/T	0.253/0.304/0.371	8.83×10^−21^	0.55 (0.49–0.63)	7.412×10^−8^	0.70 (0.09–0.10)	3.91×10^−5^	0.73 (0.63–0.85)	3.89×10^−5^	0.72 (0.10–0.12)
	rs6457617	*HLA-DQB1*	Intergenic	C/T	0.314/0.442/0.492	1.99×10^−36^	0.48 (0.42–0.54)	1.67×10^−5^	0.72 (0.10–0.12)	0.00427	0.82 (0.71-0.94)	2.68×10^−10^	0.60 (0.09–0.10)
	rs9275390	*HLA-DQB1*	Intergenic	C/T	0.454/0.177/0.253	2.61×10^−54^	2.38 (2.13–2.67)	4.793×10^−17^	1.85 (0.25–0.29)	9.70×10^−8^	0.62 (0.52–0.74)	4.45×10^−16^	0.43 (0.08–0.10)
ATA+	rs9296015	*NOTCH4*	Intergenic	A/G	0.214/0.117/0.186	0.1161	1.11 (0.97–1.27)	0.0611	1.14 (0.15–0.17)	1.14×10^−8^	0.54 (0.44–0.67)	0.000122	0.64 (0.13–0.16)
	rs3129882	*HLA-DRA*	Intron	G/A	0.430/0.631/0.440	0.2725	0.94 (0.84–1.05)	0.867	1.01 (0.11–0.12)	1.893×10^−27^	2.17 (1.88–2.50)	4.58×10^−21^	2.11 (0.30–0.35)
	rs3129763	*HLA-DQA1/DRB1*	Intergenic	A/G	0.209/0.348/0.246	0.00221	0.81 (0.71–0.93)	0.00687	0.82 (0.11–0.12)	1.474×10^−11^	1.65 (1.42–1.91)	0.000518	1.34 (0.20–0.24)
	rs987870	*HLA-DPA1/DPB1*	Intron	C/T	0.139/0.270/0.146	0.1725	0.89 (0.76–1.05)	0.525	0.94 (0.15–0.18)	2.419×10^−20^	2.09 (1.78–2.45)	1.40×10^−22^	2.67 (0.48–0.58)
	rs3135021	*HLA-DPA1/DPB1*	Intron	A/G	0.271/0.403/0.286	0.0839	0.90 (0.79–1.01)	0.463	0.95 (0.12–0.14)	1.949×10^−12^	1.66 (1.44–1.91)	2.02×10^−12^	1.81 (0.28–0.33)
	rs6901221	*HLA-DPA1/DPB1*	Intron	C/A	0.190/0.223/0.157	2.98×10^−5^	1.35 (1.17–1.55)	0.00252	1.25 (0.17–0.20)	2.542×10^−8^	1.61 (1.36–1.90)	2.55×10^−8^	1.69 (0.28–0.34)

Sample size for the ACA subgroup was 761 and for ATA was 447, while the sample size for the controls was 5,172.

**†:** Unadjusted *P* values are Mantel-Haenszel meta-analysis, GC corrected for the λ of the set, of all GWAS cohorts.

*Adjusted *P* values are logistic regression analysis adjusted for all other SNPs in the same region and the same subphenotype.

Regarding the dcSSc subtype, five non-HLA loci were found to be associated in the GWAS cohorts (Table S2 and Figure S2). Upon analyzing these five SNPs in the replication cohorts we could only replicate the association of rs11171747 in the *RPL41/ESYT1* locus (overall dcSSc *P* = 5.99×10^−8^, OR = 1.23 [1.14–1.33]) (Figure 1, Table 1 and Table S2). However, the association found in this locus was heterogeneous among cohorts (Breslow-Day *P* = 5.32×10^−9^).

### Auto-Antibodies

The observed associations in the ACA positive subgroup and lcSSc were difficult to differentiate because of substantial overlap between these two disease subgroups. In the GWAS cohorts, SNPs in *IL12RB2* and *RUNX1* genes were identified as novel non-HLA loci associated with SSc patients positive for ACA antibodies (Table S3 and Figure S3). However, none of these associations could be confirmed at the replication stage. Interestingly, the SNP rs11047102 of the *SOX5* gene, which was selected for replication due to its association with the lcSSc subgroup in the GWAS data, showed suggestive evidence of association with the ACA subgroup (*P* = 1.39×10^−7^, OR = 1.36 [1.21–1.52]) (Figure 1, Table 1 and Table S3).

In the ATA positive subgroup, four new susceptibility loci were identified in the GWAS data (Table S4 and Figure S4), none of which were confirmed in the replication phase. Since the ATA subgroup of patients has the smallest sample size, the lack of replication in any of the non-HLA locus may be due to a lower statistical power (Table S5).

### HLA Region

**Figure 2:**
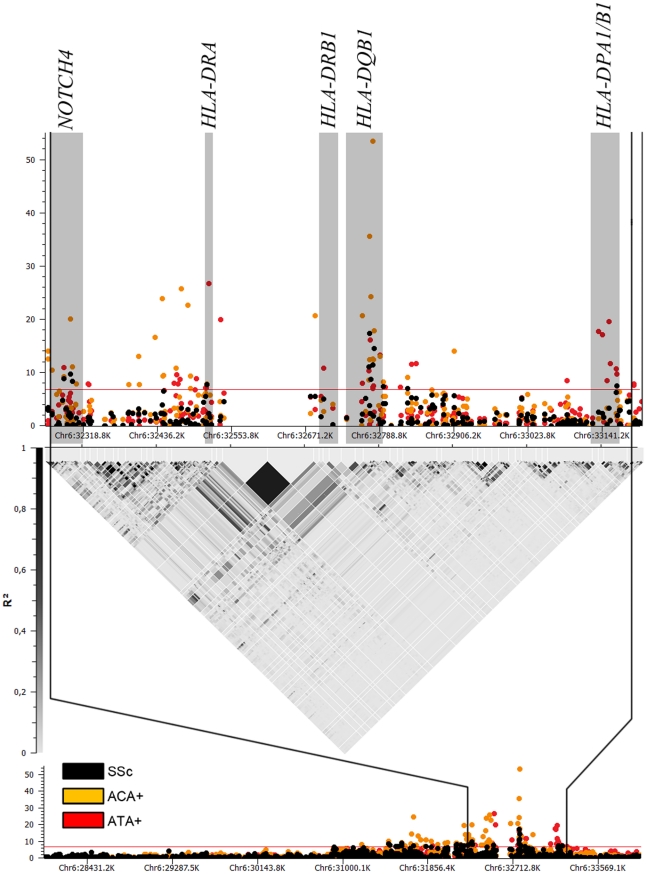
Manhattan plot showing the -log10 of the Mantel-Haenszel *P* value of all 1,112 SNPs in HLA region for the GWAS cohorts comprising 2,296 cases and 5,171 controls. Associations for the whole SSc set are in black, while associations in ACA (760 cases) and ATA (447 cases) positive subgroups are in orange and red, respectively. Loci which were independently associated according to conditional logistic regression analysis are highlighted in grey.

The associations found in the HLA region in the GWAS data set showed clear differences between SSc subgroups (Figure 1, Figure 2, and Table 2). The observed effects in the lcSSc and dcSSc subtype were similar to that of the overlapping group of patients with ACA and ATA respectively, but less significantly. Therefore, we focused the analysis on antibody subgroups only.

We observed independent genetic associations in the ACA positive subgroup in the HLA region (Table 2 and Figure 1, Table S6). The stronger independent signal was identified in the *HLA-DQB1* gene of HLA class II: SNPs rs6457617 (ACA+ *P*  =  1.99×10^−36^, OR = 0.48 [0.42–0.54]) and rs9275390 (ACA+ *P*  =  2.62×10^−54^, OR = 2.38 [2.13–2.67]). The TC allele combination (both risk alleles) showed a high association in the ACA positive subgroup (ACA+ *P*  =  7.81×10^−61^, OR = 2.48 [2.22–2.77]), being present in 45.3% of the ACA positive patients compared to 25.1% of the controls (Table 3).

**Table 3:** Allelic combination analysis of the SNPs which are in the same association locus within the HLA region for the ACA and ATA positive subgroups of SSc patients.

SSc Subphenotype	Locus	Haplotype	N (case/control)	Frequency (case/control)	*P* Value	OR (CI 95%)	SNPs
ACA	*HLA-DQB1*	TC	761/5172	0.453/0.251	7.807×10^−61^	2.48 (2.22–2.77)	rs6457617|rs9275390
		CT	761/5172	0.313/0.490	3.639×10^−38^	0.47 (0.42–0.53)	rs6457617|rs9275390
		TT	761/5172	0.234/0.259	0.0353	0.87 (0.77–0.99)	rs6457617|rs9275390
ATA	*HLA-DP*	CAC	447/5172	0.106/0.013	1.266×10^−76^	8.84 (6.72–11.63)	rs987870|rs3135021|rs6901221
		TAC	447/5172	0.019/0.012	0.0745	1.55 (0.92–2.60)	rs987870|rs3135021|rs6901221
		TGC	447/5172	0.101/0.132	0.00792	0.74 (0.59–0.92)	rs987870|rs3135021|rs6901221
		TAA	447/5172	0.265/0.256	0.562	1.05 (0.90–1.23)	rs987870|rs3135021|rs6901221
		CGA	447/5172	0.148/0.127	0.0798	1.20 (0.98–1.46)	rs987870|rs3135021|rs6901221
		TGA	447/5172	0.361/0.460	2.137×10^−8^	0.67 (0.58–0.77)	rs987870|rs3135021|rs6901221

Regarding the ATA positive subgroup, we also observed evidence of independent association in the HLA region (Table 2 and Figure 1, Table S7). We found three associations in the HLA class II region: rs3129882 in *HLA-DRA* (ATA+ *P*  =  1.89×10^−27^, OR = 2.17 [1.88–2.50]), rs3129763 in the *HLA-DQA1/DRB1* loci (ATA+ *P*  =  1.47×10^−11^, OR = 1.65 [1.42–1.91]) and four associated SNPs in the *HLA-DPA1/DPB1* region (highest association at rs987870, ATA+ *P*  =  2.42×10^−20^, OR = 2.09 [1.78–2.45]). The combination of three risk alleles in the *DPA1/DPB1* locus, CAC (ATA+ *P*  =  1.27×10^−76^, OR = 8.84 [6.72–11.63]) of the SNPs rs987870, rs3135021 and rs6901221 respectively was present in 10.6% of the ATA positive SSc patients compared to only 1.3% of the controls (Table 3).

In addition, in the HLA class III region, the *NOTCH4* gene was associated with the presence of ACA (rs443198, ACA+ *P*  =  8.84×10^−21^, OR = 0.55 [0.49–0.63]) and ATA (rs9296015, ATA+ *P*  =  1.14×10^−8^, OR = 0.54 [0.44–0.67]), independently of the HLA class II associations (Table 2 and Tables S6, S7). Interestingly, SNP rs9296015 had an opposite effect size in ACA and ATA subgroup, being exclusively associated in the ATA subgroup. These two SNPs were not in LD in Caucasian populations either from the HapMap project (r^2^ = 0.05 in CEU and r^2^ = 0.03 in TSI) or our cohorts (r^2^ = 0.1 in the combined cohorts, r^2^ = 0.11 in Spanish, r^2^ = 0.00 in German, r^2^ = 0.00 in Dutch and r^2^ = 0.01 in US), pointing to independent associations in the *NOTCH4* gene with both ACA and ATA positive subgroups. All the associations ORs found in the HLA region were consistent among the four GWAS cohorts (Tables S8, S9).

### Previously Described Genetic Associations

We wanted to investigate previously reported associations with subphenotypes or overall disease, such as *CD247, TNFSF4, STAT4, BANK1, IRF5* and *BLK* in the present study's GWAS cohorts, to further establish them as SSc (or its subphenotypes) susceptibility loci. Table S10 shows the analysis of the SNPs in the previously mentioned genes which were present in our GWAS combined panel. As expected, association previously found in these six genes was replicated. Interestingly associations previously described to be confined to one of the SSc subgroups were also replicated as in the cases of *TNFSF4* and lcSSc (lcSSc *P* = 7.70×10^−4^, OR = 1.18 [1.03–1.31]), *STAT4* and lcSSc (lcSSc *P* = 7.70×10^−8^, OR = 1.31 [1.19–1.48]), *BANK1* and dcSSc (dcSSc *P* = 0.0103, OR = 0.85 [0.75–0.96]) and *BLK* and ACA+ (ACA+ *P*  =  1.45×10^−4^, OR = 1.27 [1.12–1.44]). Furthermore association of *CD247* with SSc was more strongly represented in the lcSSc subgroup than the others (lcSSc *P*  =  2.66×10^−6^, OR = 0.81 [0.75–0.89]), although evidence of association was also found in the other subgroups. Similarly, the association found in *IRF5* was stronger in lcSSc (lcSSc *P* = 1.64×10^−10^, OR = 1.50 [1.32–1.69]), although association was also found in the dcSSc, ACA+ and ATA+ subgroups.

## Discussion

Systemic sclerosis (SSc) is a rare, severe, complex and heterogeneous rheumatic disease. Multiple lines of evidence suggest that genetic factors may underlie not only SSc susceptibility but also the predisposition to develop specific clinical phenotypes such as lcSSc, dcSSc subtypes and the presence of SSc-specific auto-antibodies. The discovery of genetic variants associated with specific clinical manifestations of the disease will lead to new insights regarding pathogenesis and may open novel avenues of therapy that can be targeted to specific subsets.

The aim of this study was to assess the genetic component involved in four different SSc clinical and auto-antibody subphenotypes through an analysis of our previous genome-wide association study (GWAS) data stratified for these disease subphenotypes, together with a large, new replication study.

We have identified an association of the *NOTCH4* gene with both ACA and ATA positive subgroups independent of the HLA associations. This gene is located in the MHC and encodes a transmembrane protein which plays a role in a variety of developmental processes by controlling cell fate decisions. Interestingly, *NOTCH4* has been implicated in the pathways by which TGF-β induces pulmonary fibrosis [24], one of the most severe clinical manifestations of SSc [25], [26]. The Notch signaling pathway also controls key functions in vascular smooth muscle and endothelial cells which may be particularly relevant to the microvascular damage seen in SSc [27]. Genetic variants in *NOTCH4* also have been previously associated, independently from HLA genes or alleles, with other autoimmune disorders like diabetes type 1 [28], rheumatoid arthritis [29] and alopecia areata [30], [31].

Additionally, through the analysis of the largest SSc case/control cohort reported to date we identified three new susceptibility loci (*IRF8*, *SOX5* and *GRB10*), outside the HLA/MHC region, implicated in genetic predisposition to different SSc subphenotypes, in addition to other suggestive loci.

Type I and II interferons (IFN) are well known immunomodulators which can also regulate collagen production. Furthermore, they are believed to play a key role in the pathogenesis of SSc and other autoimmune diseases [32]–[34]. Interestingly, we found a strong association of the *IRF8* gene with the lcSSc subtype and the ACA positive subgroup. *IRF8* modulates TLR signaling and may contribute to the crosstalk between IFN-γ and TLR signal pathways, thus acting as a link between innate and adaptive immune responses [35]. *IRF8* also has been demonstrated to be a key factor in B cell lineage specification, commitment and differentiation [36]. In addition, *IRF8* has been associated with another autoimmune disease, multiple sclerosis [37], although the SNP associated with multiple sclerosis (rs17445836) was not present in our study. Nevertheless, both variants are in medium LD in the CEU population of the HapMap project (r^2^ = 0.51) and both associations have a protective OR for the minor allele; pointing to a dependence in the associations found in these two diseases.

The most prominent SSc specific auto-antibodies, ACA and ATA, are associated with the lcSSc and dcSSc clinical subsets, respectively [19]. The lcSSc subtype greatly overlaps with the ACA positive subgroup of patients (almost all ACA positive patients belonged to the lcSSc subtype). Similarly, the dcSSc subtype overlaps with the ATA positive group of patients. Therefore, it is difficult to determine whether some of the observed associations specifically belonged to one of the four subgroups. Such is the case of the association found with the *SOX5* gene. In the GWAS data, *SOX5* was associated with lcSSc as well as with the ACA positive subgroup, although the association with the lcSSc subtype was stronger than that in the ACA positive subgroup. Upon completion of the replication study with the resultant increase in statistical power, we were able to determine that the *SOX5* gene was indeed a risk factor for the ACA positive group at the genome wide significance level, but not for lcSSc. The *SOX5* gene encodes a member of the SOX (*SRY*-related HMG-box) family of transcription factors involved in the regulation of embryonic development, in the determination of cell fate, as well as in chondrogenesis [38].

Conversely *SOX5*, together with *SOX6* and *SOX9*, can induce many cellular types (including melanocytes and bone marrow stem cells) into the chondrogenic pathway, leading to expression of *COL2A1* and the formation of cartilage [38], [39]. As stated above, IFN type I and II are inhibitors of collagen production and chondrogenesis; more precisely IFN-γ (type II IFN) inhibits the *COL2A1* gene which is one of the main downstream genes in the chondrogenesis pathway [40]. Taken all together, *IRF8* (part of the interferon pathway and induced by IFN-γ [41]) and *SOX5* may be affecting the formation of the extra-cellular matrix through *COL2A1* in the skin and other organs of SSc patients.

We also identified an association of the *GRB10* gene with the lcSSc subtype; *GRB10* codes for an adaptor protein known to interact with a number of tyrosine kinase receptors and signaling molecules and has a potential role in apoptosis regulation [42].

In dcSSc patients, the only observed genome wide significant association was with the *RPL41/ESYT1* locus, although this association was heterogeneous among the investigated populations, probably due to lower statistical power in this smaller group. Three genes are relevant to this locus: *RPL41*, a ribosomal protein not considered to be related to the immune system; *ZC3H10*, a zinc finger protein related to tumour growth; and *ESYT1*, a synaptotagmin-like protein of unknown function. Although none of these genes has a suggestive role in the pathogenesis of SSc *a priori*, further studies are needed to investigate this intriguing finding.

Since most genes in the HLA region are implicated in the regulation of the immune system, it is not surprising that the HLA-association with SSc is primarily related to auto-antibody expression. We found different patterns of independent association for the two major SSc auto-antibody subgroups across the HLA class II region. Both genetic markers located in the *HLA-DQB1* locus were associated with the presence of ACA auto-antibodies in SSc patients. The allelic combination of these SNPs tags the described association of HLA-DQB1*0501 with the ACA positive subgroup of the disease [22], [43]. The associations within the HLA region in the ATA positive subgroup are more complex: SNP rs3129763 (located near *HLA-DRB1*) tags the association of HLA-DRB1*1104, which has been described to be associated with the whole disease [22]. Furthermore, the haplotype in the *HLA-DPB1* region described in Table 3, tags the HLA-DPB1*1301 also previously described [3], [22]. Interestingly, the remaining independent association observed, rs3129882, is found within the HLA-DRA gene, which is much less polymorphic than the other HLA genes already mentioned; nevertheless, the association found in this SNP is tagging through the extensive LD structure of the MHC region the association of some aminoacidic positions in the nearby *HLA-DQB1* gene, which has not been previously reported to be associated with the ATA positive subgroup of SSc.

In summary, taking advantage of our GWAS data and a large replication cohort, we have identified three new non-HLA loci associated with subphenotypes of SSc: *GRB10*, *IRF8*, and *SOX5*. In addition, we shed light on HLA associations with this disease, establishing different patterns of independent association in the ACA and ATA positive subgroups. Our findings provide evidence for genetic heterogeneity underlying the clinical and especially autoantibody subtypes of SSc. These findings may prompt reconsideration of the current classification of SSc patients; provide insight into pathogenetic pathways differing among subphenotypes, especially specific auto-antibody subgroups, and lead to novel therapeutic targets for this devastating autoimmune disease.

## Materials and Methods

### Subjects

For the GWAS analysis, a total of 2,296 Caucasian SSc patients and 5,171 Caucasian healthy controls were recruited through an international collaborative effort in the United States of America (USA), Spain, Germany and The Netherlands. The North American cases (initial n = 1,678; after applying quality control criteria, n = 1,486; 179 men, 1,307 women; mean age = 54.5 (median, 55.0); SD = 12.9) were recruited from May, 2001 to December, 2008 from three U.S. sources: the Scleroderma Family Registry and DNA Repository and the Center of Research Translation in Scleroderma at The University of Texas (UT) Health Science Center-Houston, The Johns Hopkins University Medical Center and the Fred Hutchinson Cancer Research Center, each enrolling patients from a US-wide catchment area. The initial European SSc cases came from previously established nationally representative collections of 380 Spanish, 288 German and 190 Dutch patients with SSc. As control populations, healthy unrelated individuals of Spanish (initial n = 414), German (initial n = 678) and Dutch (initial n = 643) origin were included in the study as well as 3478 controls from across the US collected as non-cancer controls for GWAS studies of breast and prostate cancers in the Cancer Genetic Markers of Susceptibility (CGEMS) studies [44], [45] (http://cgems.cancer.gov/data_access.html).

In the second replication phase, a large independent replication cohort, consisting of 3,175 SSc patients and 4,971 healthy controls of Caucasian ancestry, were collected from Belgium, Spain, The Netherlands, Germany, Italy, Norway, Sweden, UK and the USA. Details on the investigated populations are provided in the Table S11.

All cases met the American College of Rheumatology preliminary criteria for the classification of SSc [46]. Furthermore, patients were classified according to the extent of skin involvement into limited (lcSSc) or diffuse (dcSSc) forms [17], [47]. In addition, the presence of SSc specific auto-antibodies, anti-topoisomerase I (ATA, Anti-Scl70) and anti-centromere (ACA) was assessed by passive immunodiffusion against calf thymus extract (Inova Diagnostics, San Diego, CA, USA) and indirect immunoflourescence of HEp-2 cells (Antibodies Inc, Davis, CA, USA), respectively, in a total of 5,229 and 5,238 SSc patients respectively. Auto-antibodies to RNA Polymerase III are also considered to be characteristic of SSc, but testing for this antibody is not widely available and since results were not known in almost two-thirds of our cases, this analysis was not done [18], [19]. The distribution of SSc patients among these disease subsets is summarized in Table S11.

Collection of blood samples and clinical information from case and control subjects was undertaken with informed consent and relevant ethical review board approval from each contributing centre in accordance with the tenets of the Declaration of Helsinki.

Most of the individuals included in this study, GWAS and replication cohorts, have been analyzed in a previous study [15] but novel genotypes were generated in the replication cohorts for phenotype associated SNPs found in the GWAS, expanding the scope of the study.

### SNP Selection for Replication

Our goal was to examine any novel genetic association specific for each subset rather than overall disease. Although partial overlapping exists between lcSSc and ACA+ subgroups, and dcSSc and ATA+ subgroups; we wanted to assess whether association found in overlapped groups belonged to a subtype or an auto-antibody positive group. With that purpose we selected SNPs from the GWAS data based on the following criteria:

- First, we selected all SNPs with a *P* value of 1×10^−5^ or lower in each of the four considered SSc subgroups (*i.e.* lcSSc, dcSSc, ACA+ and ATA+) of the four GWAS cohorts (*i.e.* US, Spain, Netherlands and Germany).
- Since one aim of this study was to find novel genetic associations, we then ruled out every genetic association previously described in SSc (*e.g. STAT4*, *IRF5* and the HLA region).
- To select subphenotype specific signals, we excluded all SNPs with *P* values of the same order of magnitude or lower in the opposite group, *i.e.* lcSSc versus dcSSc and ACA-positive versus ATA-positive.
- Finally we selected from each remaining region the best independent association (determined by conditional logistic regression) from the GWAS data.

This resulted in the selection of 18 non-HLA SNPs (7 for lcSSc, 5 for dcSSc, 2 for ACA+, and 4 for ATA+) as shown in Tables S1, S2, S3, S4, corresponding to lcSSc, dcSSc, ACA and ATA positive patients respectively.

### Genotyping

The GWAS genotyping of the SSc cases and controls was performed as follows: the Spanish SSc cases and controls together with Dutch and German SSc cases was performed at the Department of Medical Genetics of the University Medical Center Utrecht (The Netherlands) using the commercial release Illumina HumanCNV370K BeadChip, which contains 300,000 standard SNPs with an additional 52,167 markers designed to specifically target nearly 14,000 copy number variant regions of the genome, for a total of over 370,000 markers. Genotype data for Dutch and German controls were obtained from the Illumina Human 550K BeadChip available from a previous study. The SSc case group from the United States was genotyped at Boas Center for Genomics and Human Genetics, Feinstein Institute for Medical Research, North Shore Long Island Jewish Health System using the Illumina Human610-Quad BeadChip. CGEMS and Illumina iControlDB controls were genotyped on the Illumina Hap550K-BeadChip.

SNPs selected for the replication phase were genotyped in the replication cohorts using Applied Biosystems' TaqMan SNP assays on ABI Prism 7900 HT real-time thermocyclers. Markers with call rates of 95% or less were excluded, as were markers whose allele distributions deviated strongly from Hardy-Weinberg (HW) equilibrium in controls (P<10^−3^).

### Data Imputation

Imputation was performed in the GWAS cohorts in order to gain genome coverage for the SNP selection. Imputation was performed with IMPUTE software 1.00 as previously described [48], using as reference panels the CEU and TSI HapMap populations. However, SNP imputation did not show any new independent SNP associated at *P*<10^−5^ in the four subphenotypes considered. The imputed GWAS data in the four subphenotypes is shown in Figure S5.

### Statistical Analysis

Data in the SSc GWAS cohorts was filtered as follows: Using Plink, we identified and excluded pairs of genetically related subjects or duplicates and excluded the genetic-pair members with lower call rates. To identify individuals who might have non–western European ancestry, we merged our case and control data with the data from the HapMap Project (60 western European (CEU), 60 Nigerian (YRI), 90 Japanese (JPT) and 90 Han Chinese (CHB) samples). We used principal component analysis as implemented in HelixTree (see Text S2), plotting the first two principal components for each individual. All individuals who did not cluster with the main CEU cluster (defined as deviating more than 4 standard deviations from the cluster centroids) were excluded from subsequent analyses. Additionally, we excluded individuals with low call rates (11 individuals from the US group, 24 from the Spanish, 1 from the German and 1 from the Dutch), relatedness (50 from the US group, 2 from the Spanish, 1 from the German and 1 from the Dutch), non-European ancestry (42 from the US group, 5 from the Spanish, 6 from the German and 4 from the Dutch) and inconsistent gender (83 from the US group, 2 from the Spanish, 2 from the German and 2 from the Dutch). Then we filtered for SNP quality, removing SNPs with a genotyping success call rate < 98% and those showing MAF < 1%. Deviation of the genotype frequencies in the controls from those expected under Hardy-Weinberg equilibrium was assessed by a χ^2^ test or Fisher's exact test when an expected cell count was < 5. SNPs strongly deviating from Hardy-Weinberg equilibrium (*P*<10^−5^) were eliminated from the study. For the combined analysis of the four datasets, the same quality controls per individual and per SNP were applied with the exception of the Hardy-Weinberg equilibrium (HWE) requirement. The genotyping success call rate on the merged dataset after all these quality filters were applied was 99.83% in the GWAS cohorts.

The replication cohorts were filtered as follows: all individuals with a SNP success call rate below 0.95 were excluded, SNPs with a per individual success call rate below 0.95 were excluded, SNPs with a HWE comparison *P* value below 0.001 in controls were excluded and SNPs with a MAF below 0.01 were also excluded. As a result, 18 SNPs selected for replication all were in HWE (*P* value > 0.001) and the overall genotype successful call rate was 96.61% and all SNPs individually had a successful call rate greater than 95%.

We performed power calculations for GWAS and replication cohorts for the whole dataset and the clinical/auto-antibodies subphenotypes according to Skol *et al.* [49] (Table S5). The significance level for these calculations was set at 5×10^-8^.

*χ^2^* tests were performed for allelic model for significant differences between cases and controls. Derived *P* values for the replication cohorts were not adjusted. All nine replication cohorts were jointly analyzed conducting Cochran-Mantel-Haenszel (CMH) tests to control for population differences. A threshold meta-analysis *P* value of <0.05 for the replication phase was considered significant. We also conducted CMH meta-analysis of all the nine replication cohorts and the four cohorts previously included in the GWAS, considering a *P* value lower than 5×10^−8^ as significant. Furthermore, *P* values in the range 5×10^−8^ to 5×10^−6^ were considered as suggestive associations. In all tests, odds ratios (OR) were calculated according to Woolf's method. We also applied Breslow-Day (BD) tests for all meta-analyses to check for heterogeneity in association among the investigated populations, and all associations with a *P*<0.05 in BD analysis were considered heterogeneous.

Due to the partial overlapping of the lcSSc and dcSSc subgroups with ACA+ and ATA+ subgroups, respectively, we wanted to test whether an association found in both overlapping groups belonged to one or the other specifically. With that purpose, all the associations in the present study claimed to belong to a group were tested for association in the correlated group (*e.g.* ACA associations were tested in lcSSc and vice versa) to look for the best *P* value. In addition, ACA and ATA hits were tested in lcSSc-ACA- and dcSSc-ATA-, respectively, to ensure group specific associations. Also, lcSSc and dcSSc were tested in ACA+-non-lcSSc and ATA+-non-dcSSc with the same purpose.

To determine independent associations in the HLA region, conditional logistic regression was carried out for all associated SNPs in the complete SSc group and the ACA and ATA positive subgroups. This analysis was carried out as implemented in Plink software, conditioning each SNP association to each of the other significantly associated (*P*<5×10^−7^) SNPs in the corresponding LD block, controlling for the presence of the four populations as covariates. All SNPs which remained significant after conditioning were considered independent associations. All haplotype analysis was performed using Haploview software, defining the blocks by confidence intervals [50]. We only analyzed haplotypes or allelic combinations with frequencies of 1% and above.

Statistical analyses were undertaken using R (v2.6), Stata (v8), Plink (v1.07) [51] and HelixTree's SNP & Variation Suite (v7.3.0) software (see Text S2).

### Web Resources

Plink software:

http://pngu.mgh.harvard.edu/purcell/plink/

SVS HelixTree software:

http://www.goldenhelix.com/SNP_Variation/HelixTree/index.html

Stata software:

http://www.stata.com/

R Statistical Package:

http://www.r-project.org/

Haploview:

http://www.broadinstitute.org/scientific-community/science/programs/medical-and-population-genetics/haploview/haploview

## Supporting Information

Figure S1 Manhattan plot and QQ plot showing the -log10 of the Mantel-Haenszel *P* value of all 279,621 SNPs in the lcSSc individuals of the GWAS cohorts comprising 1,400 cases and 5,171 controls. All *P* values are GC corrected, and λ was 1.058.
(TIF)

Figure S2 Manhattan plot and QQ plot showing the -log10 of the Mantel-Haenszel *P* value of all 279,621 SNPs in the dcSSc individuals of the GWAS cohorts comprising 740 cases and 5,171 controls. All *P* values are GC corrected, and λ was 1.034.
(TIF)

Figure S3 Manhattan plot and QQ plot showing the -log10 of the Mantel-Haenszel *P* value of all 279,621 SNPs in the ACA positive individuals of the GWAS cohorts comprising 761 cases and 5,171 controls. All *P* values are GC corrected, and λ was 1.050.
(TIF)

Figure S4 Manhattan plot and QQ plot showing the -log10 of the Mantel-Haenszel *P* value of all 279,621 SNPs in the ATA positive individuals of the GWAS cohorts comprising 447 cases and 5,171 controls. All *P* values are GC corrected, and λ was 1.061.
(TIF)

Figure S5 Manhattan plot showing the analysis in the GWAS cohorts imputed data. The different subphenotypes considered are represented in different colors.
(TIF)

Table S1 Analysis for GWAS cohorts, replication cohorts and combined analysis for all non-HLA, non-previously described associations with lcSSc subtype of the disease. †*P* values for GWAS cohorts are Mantel-Haenszel meta-analysis GC corrected according to the set λ and in the replication and combined analysis Mantel-Haenszel meta-analysis *P* value. ‡*P* value for the totality of the SSc patients, in the case of GWAS cohorts GC corrected according to the set λ, and in replication and combined analysis Mantel-Haenszel meta-analysis *P* value.
(DOC)

Table S2 Analysis for GWAS cohorts, replication cohorts and combined analysis for all non-HLA, non-previously described associations with dcSSc subtype of the disease. †*P* values for GWAS cohorts are Mantel-Haenszel meta-analysis GC corrected according to the set λ and in the replication and combined analysis Mantel-Haenszel meta-analysis *P* value. ‡*P* value for the totality of the SSc patients, in the case of GWAS cohorts GC corrected according to the set λ, and in replication and combined analysis Mantel-Haenszel meta-analysis *P* value. *Association in rs11171747 had a significant BD *P* value, thus making them heterogenic associations among populations.
(DOC)

Table S3 Analysis for GWAS cohorts, replication cohorts and combined analysis for all non-HLA, non-previously described associations with ACA positive subgroup of the disease. †*P* values for GWAS cohorts are Mantel-Haenszel meta-analysis GC corrected according to the set λ and in the replication and combined analysis Mantel-Haenszel meta-analysis *P* value. ‡*P* value for the totality of the SSc patients, in the case of GWAS cohorts GC corrected according to the set λ, and in replication and combined analysis Mantel-Haenszel meta-analysis *P* value. *Association in rs3790567 had a significant BD *P* value, thus making them heterogeneous associations among populations.
(DOC)

Table S4 Analysis for GWAS cohorts, replication cohorts and combined analysis for all non-HLA, non-previously described associations with ATA positive subgroup of the disease. †*P* values for GWAS cohorts are Mantel-Haenszel meta-analysis GC corrected according to the set λ and in the replication and combined analysis Mantel-Haenszel meta-analysis *P* value. ‡*P* value for the totality of the SSc patients, in the case of GWAS cohorts GC corrected according to the set λ, and in replication and combined analysis Mantel-Haenszel meta-analysis *P* value.
(DOC)

Table S5 Power calculations and genomic inflation factors (λ) in the whole SSc cohorts (GWAS and replication) and the lcSSc, dcSSc, ACA and ATA positive subphenotypes. 5×10^−8^ was used as significance threshold.
(DOC)

Table S6 Conditional logistic regression analysis of all the independently associated SNPs in the HLA region in the ACA positive patients. †*P* values for Mantel-Haenszel meta-analysis GC corrected according to the set λ.
(DOC)

Table S7 Conditional logistic regression analysis of all the independently associated SNPs in the HLA region in the ATA positive patients. †*P* values for Mantel-Haenszel meta-analysis GC corrected according to the set λ.
(DOC)

Table S8 Independent associations found in the HLA region in the ACA positive subgroup of patients in the separate four GWAS cohorts. †Uncorrected χ^2^ *P* value of each separated cohort.
(DOC)

Table S9 Independent associations found in the HLA region in the ATA positive subgroup of patients in the separate four GWAS cohorts. †Uncorrected χ^2^ *P* value of each separated cohort.
(DOC)

Table S10 Previously described genetic associations with SSc subphenotypes which were present in the present study's GWAS panel of SNPs. A total of 2,296 SSc cases and 5,172 controls were included in this analysis. The SSc cases included 1,400 lcSSc individuals, 740 dcSSc individuals, 761 ACA+ individuals and 447 ATA+ individuals. Best *P* value in each subgroup for each SNP is in bold. Chr. Chromosome. † Uncorrected Mantel-Haenszel Meta-analysis *P* value of the four GWAS cohorts.
(DOC)

Table S11 Composition and size of all the populations used in the study for the considered features of the disease.
(DOC)

Text S1 Members of the Spanish Scleroderma Group.
(DOC)

Text S2 URLs. Internet Uniform Resource Locator (URL) for each of the software packages used in this study.
(DOC)
