# Correction: Identification of Novel Genetic Markers Associated with Clinical Phenotypes of Systemic Sclerosis through a Genome-Wide Association Strategy

**DOI:** 10.1371/annotation/3aeebb2e-64e5-4548-8d65-1f2d5dfeb073

**Published:** 2011-08-25

**Authors:** Olga Gorlova, Jose-Ezequiel Martin, Blanca Rueda, Bobby P. C. Koeleman, Jun Ying, Maria Teruel, Lina-Marcela Diaz-Gallo, Jasper C. Broen, Madelon C. Vonk, Carmen P. Simeon, Behrooz Z. Alizadeh, Marieke J. H. Coenen, Alexandre E. Voskuyl, Annemie J. Schuerwegh, Piet L. C. M. van Riel, Marie Vanthuyne, Ruben van 't Slot, Annet Italiaander, Roel A. Ophoff, Nicolas Hunzelmann, Vicente Fonollosa, Norberto Ortego-Centeno, Miguel A. González-Gay, Francisco J. García-Hernández, María F. González-Escribano, Paolo Airo, Jacob van Laar, Jane Worthington, Roger Hesselstrand, Vanessa Smith, Filip de Keyser, Fredric Houssiau, Meng May Chee, Rajan Madhok, Paul G. Shiels, Rene Westhovens, Alexander Kreuter, Elfride de Baere, Torsten Witte, Leonid Padyukov, Annika Nordin, Raffaella Scorza, Claudio Lunardi, Benedicte A. Lie, Anna-Maria Hoffmann-Vold, Øyvind Palm, Paloma García de la Peña, Patricia Carreira, Spanish Scleroderma Group, John Varga, Monique Hinchcliff, Annette T. Lee, Pravitt Gourh, Christopher I. Amos, Frederick M. Wigley, Laura K. Hummers, J. Lee Nelson, Gabriella Riemekasten, Ariane Herrick, Lorenzo Beretta, Carmen Fonseca, Christopher P. Denton, Peter K. Gregersen, Sandeep Agarwal, Shervin Assassi, Filemon K. Tan, Frank C. Arnett, Timothy R. D. J. Radstake, Maureen D. Mayes, Javier Martin

There is an error in Laura K. Hummers' affiliation. Dr. Hummers should be affiliated with The Johns Hopkins University Medical Center, Baltimore, Maryland, United States of America. 

