# Supplementary figures and images for: Identification of Novel Genetic Markers Associated with Clinical Phenotypes of Systemic Sclerosis through a Genome-Wide Association Strategy
Source: PLoS Genet. 2011 Jul 14;7(7):e1002178. doi: 10.1371/journal.pgen.1002178 (PMC3136437; doi:10.1371/journal.pgen.1002178)

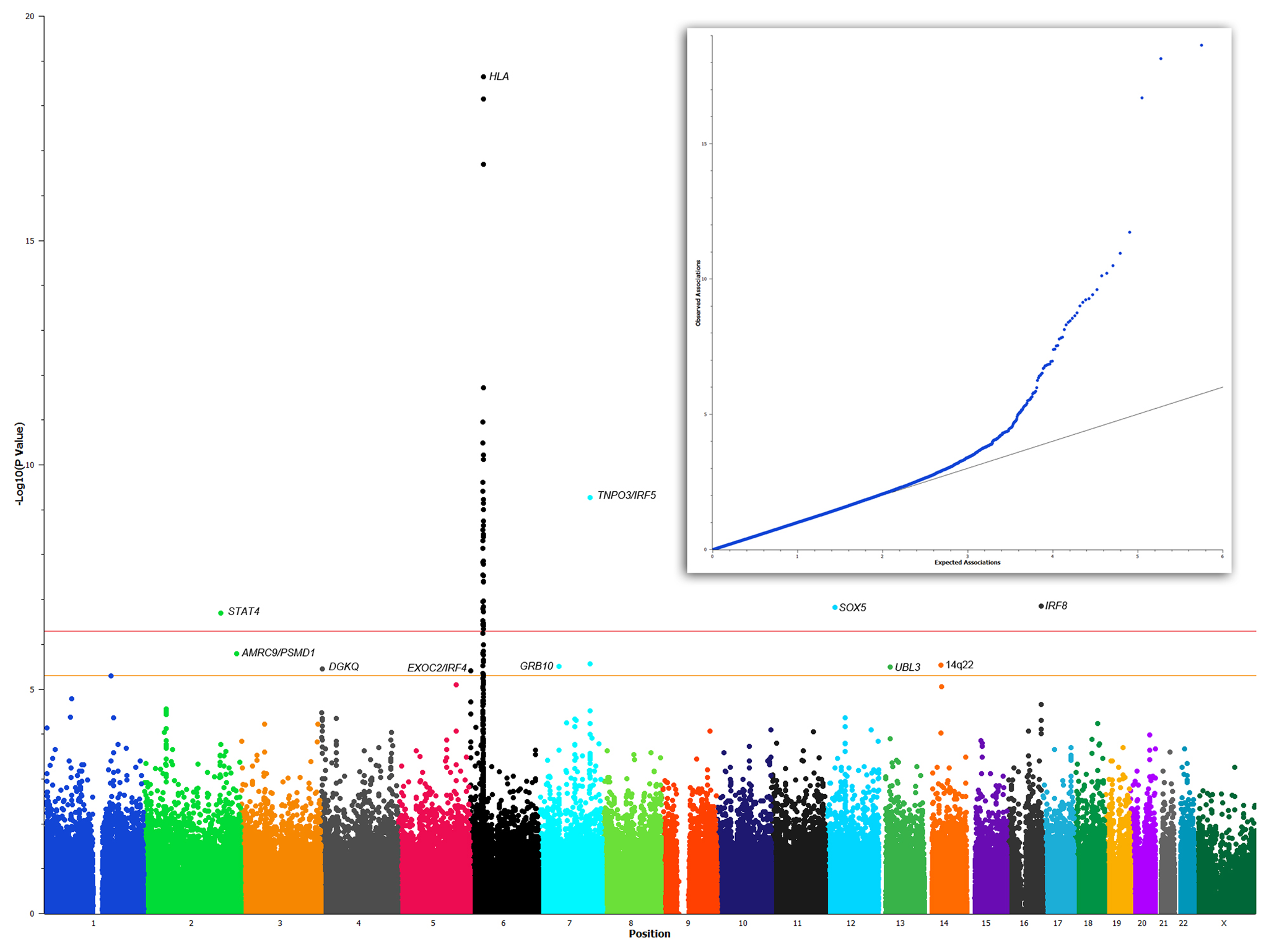

Supplement: Figure S1 — Manhattan plot and QQ plot showing the -log10 of the Mantel-Haenszel P value of all 279,621 SNPs in the lcSSc individuals of the GWAS cohorts comprising 1,400 cases and 5,171 controls. All P values are GC corrected, and λ was 1.058. (TIF) [file pgen.1002178.s001.tif]

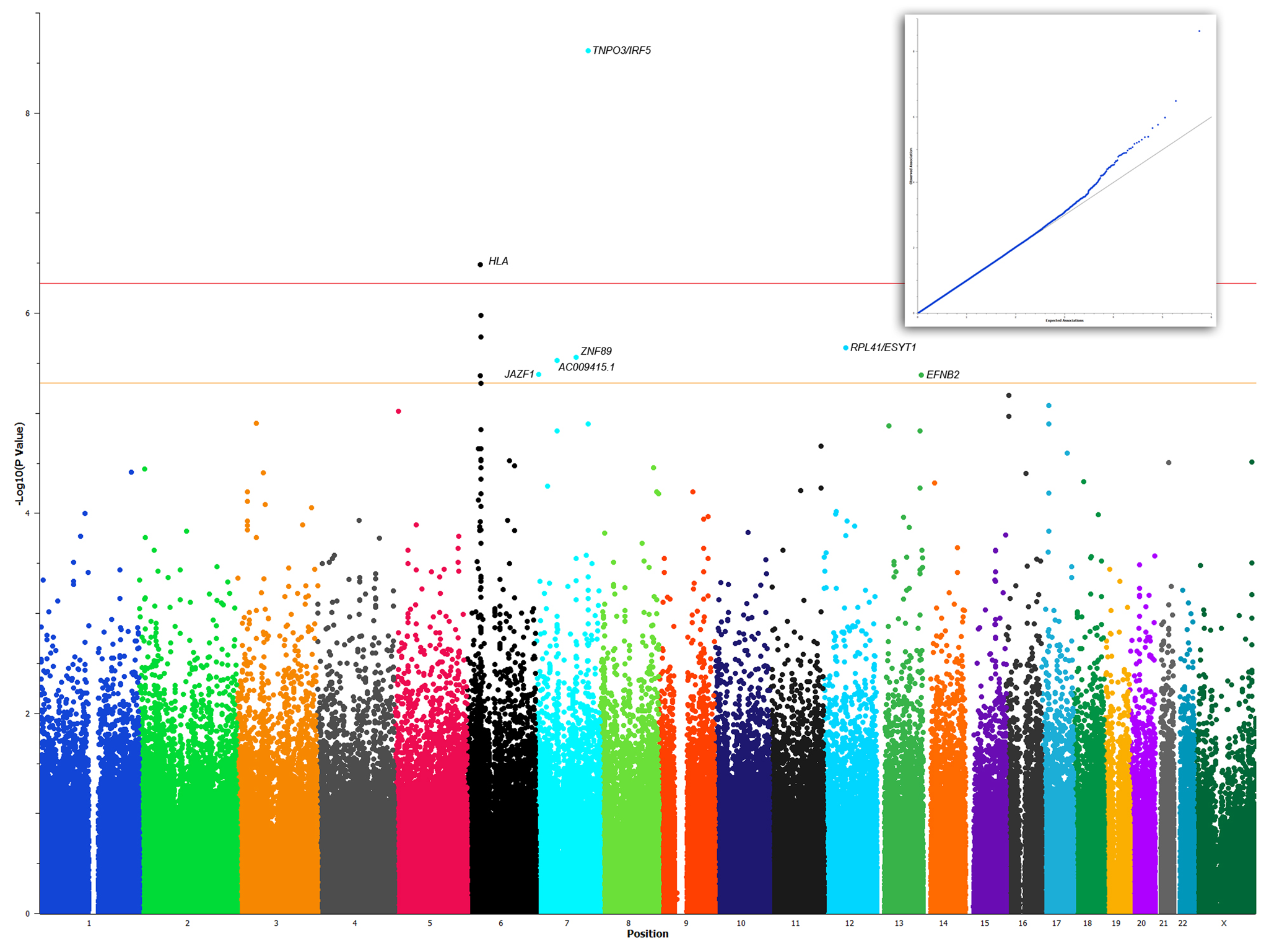

Supplement: Figure S2 — Manhattan plot and QQ plot showing the -log10 of the Mantel-Haenszel P value of all 279,621 SNPs in the dcSSc individuals of the GWAS cohorts comprising 740 cases and 5,171 controls. All P values are GC corrected, and λ was 1.034. (TIF) [file pgen.1002178.s002.tif]

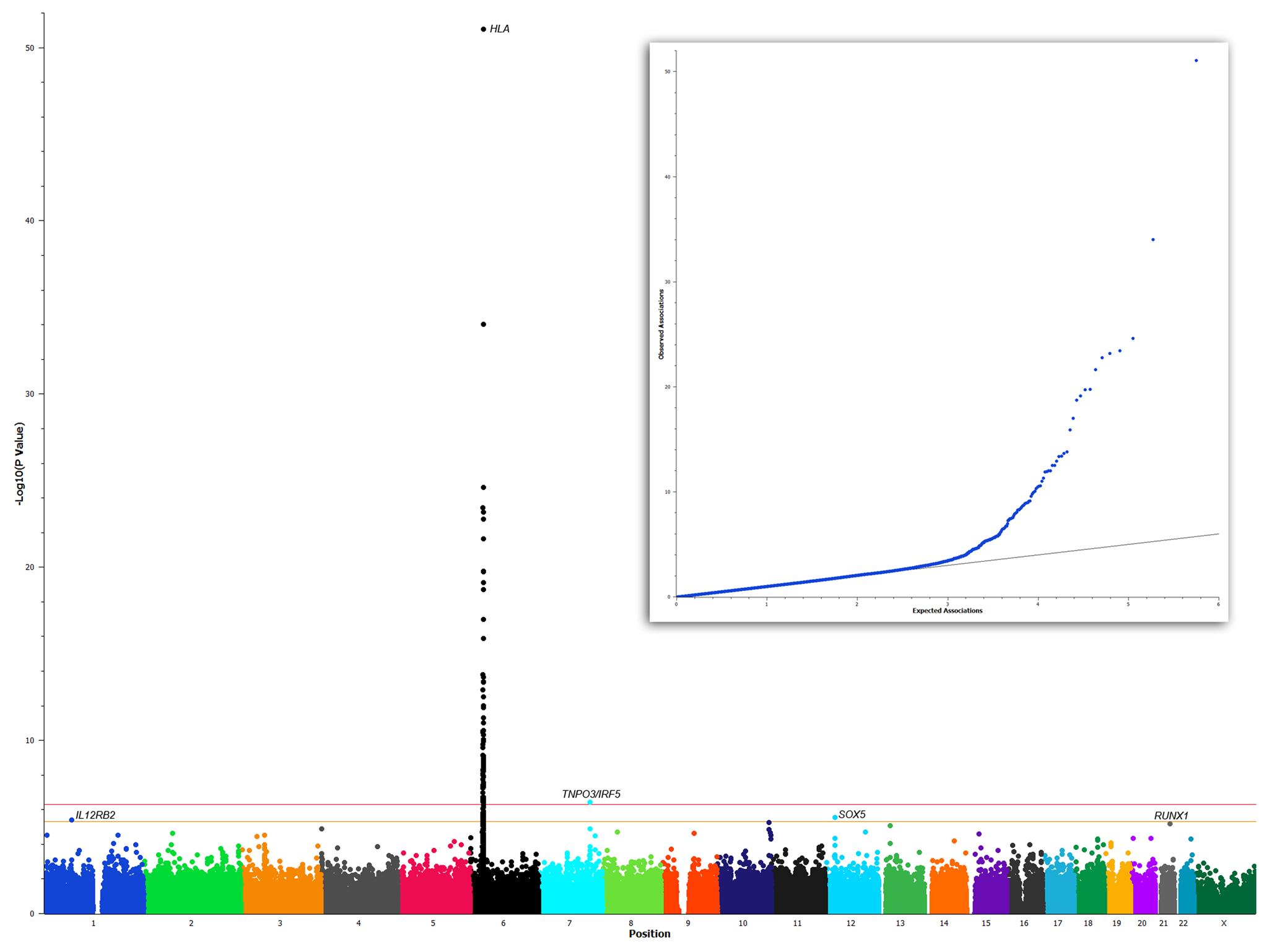

Supplement: Figure S3 — Manhattan plot and QQ plot showing the -log10 of the Mantel-Haenszel P value of all 279,621 SNPs in the ACA positive individuals of the GWAS cohorts comprising 761 cases and 5,171 controls. All P values are GC corrected, and λ was 1.050. (TIF) [file pgen.1002178.s003.tif]

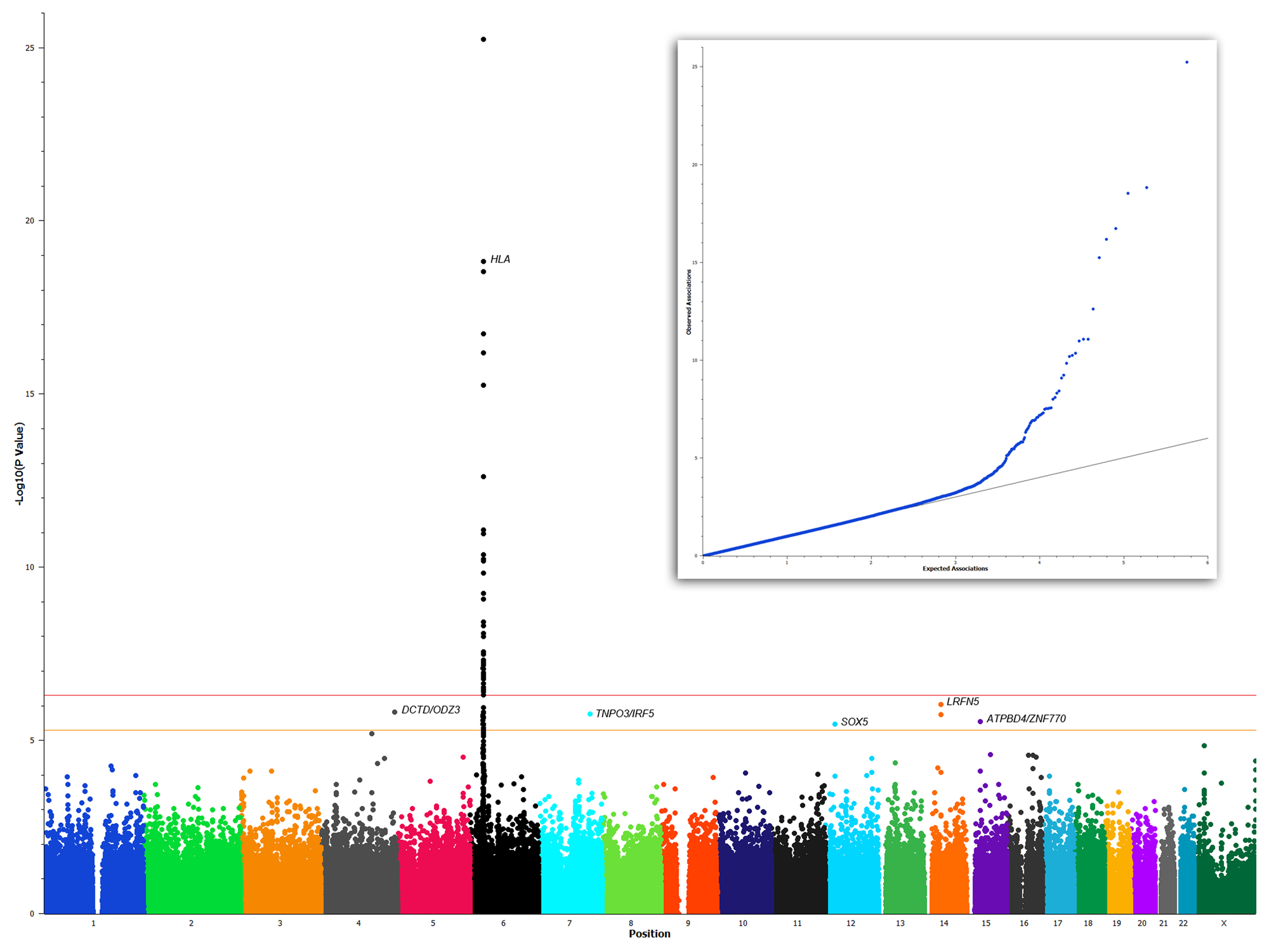

Supplement: Figure S4 — Manhattan plot and QQ plot showing the -log10 of the Mantel-Haenszel P value of all 279,621 SNPs in the ATA positive individuals of the GWAS cohorts comprising 447 cases and 5,171 controls. All P values are GC corrected, and λ was 1.061. (TIF) [file pgen.1002178.s004.tif]

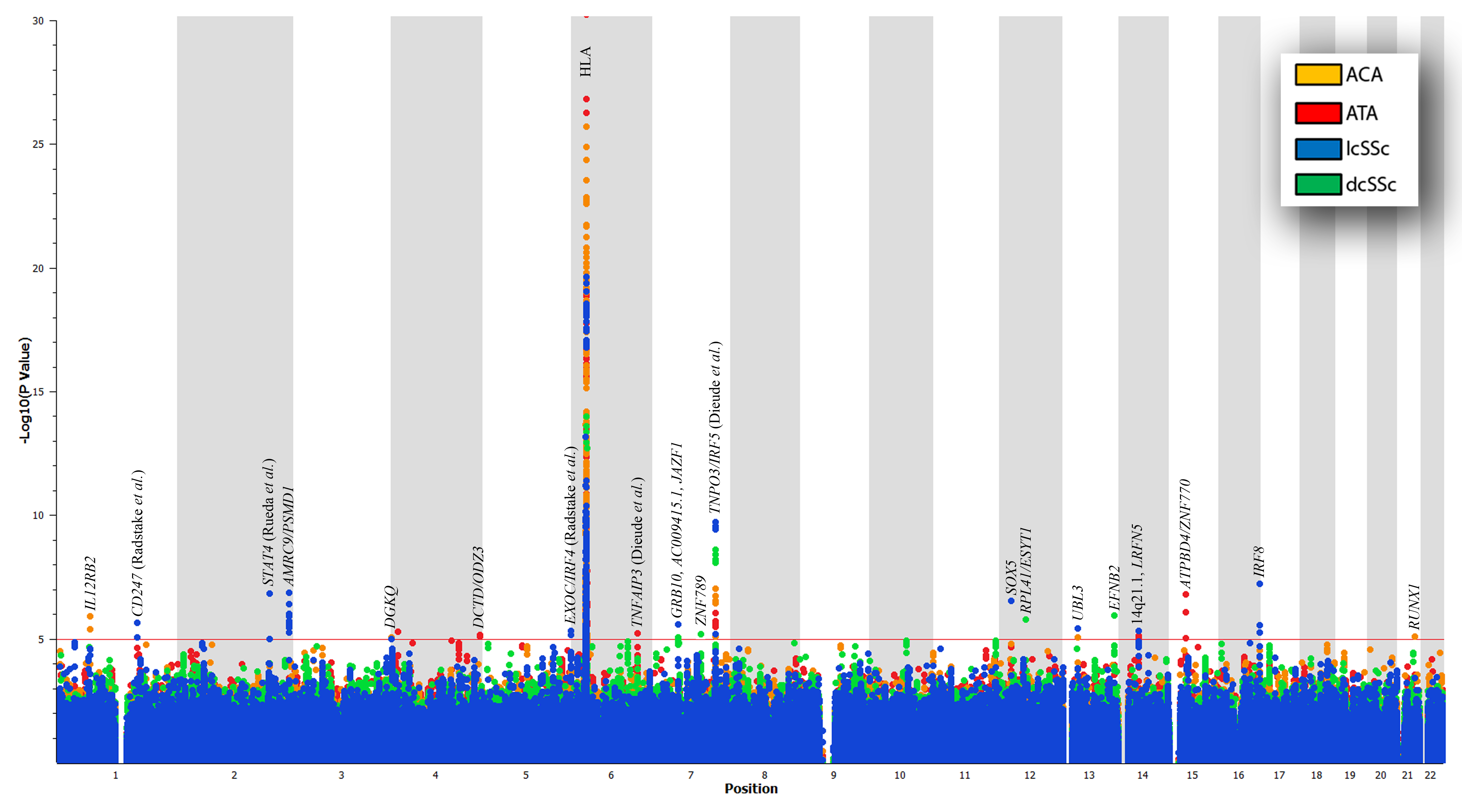

Supplement: Figure S5 — Manhattan plot showing the analysis in the GWAS cohorts imputed data. The different subphenotypes considered are represented in different colors. (TIF) [file pgen.1002178.s005.tif]
