# Supplementary material for: Identification of Novel Genetic Markers Associated with Clinical Phenotypes of Systemic Sclerosis through a Genome-Wide Association Strategy
Source: PLoS Genet. 2011 Jul 14;7(7):e1002178. doi: 10.1371/journal.pgen.1002178 (PMC3136437; doi:10.1371/journal.pgen.1002178)
Supplement: Table S1 — Analysis for GWAS cohorts, replication cohorts and combined analysis for all non-HLA, non-previously described associations with lcSSc subtype of the disease. †P values for GWAS cohorts are Mantel-Haenszel meta-analysis GC corrected according to the set λ and in the replication and combined analysis Mantel-Haenszel meta-analysis P value. ‡P value for the totality of the SSc patients, in the case of GWAS cohorts GC corrected according to the set λ, and in replication and combined analysis Mantel-Haenszel meta-analysis P value. (DOC) [file pgen.1002178.s006.doc]

| Chr. | Gene | SNP | Base Pair | Location | Change | Stage | N (case/control) | MAF (case/control) | *P* value† | OR (CI 95%) | Full set *P*‡ | dcSSc *P*† | ACA+ *P*† |
| --- | --- | --- | --- | --- | --- | --- | --- | --- | --- | --- | --- | --- | --- |
| 12p12.1 | *SOX5* | rs11047102 | 23,837,413 | Intronic | T/C | GWAS | 1400/5172 | 0.132/0.097 | 1.49x10^-7^ | 1.43 (1.26-1.63) | 1.36x10^-6^ | 0.222 | 1.03x10^-5^ |
|  |  |  |  |  |  | Replication | 1960/4971 | 0.108/0.102 | 0.244 | 1.08 (0.95-1.23) | 0.162 | 0.351 | 0.00291 |
|  |  |  |  |  |  | Combined | 3360/10143 | 0.118/0.099 | 5.11x10^-6^ | 1.24 (1.13-1.35) | 7.52x10^-6^ | 0.127 | 1.39x10^-7^ |
| 16q24.1 | *IRF8* | rs11642873 | 84,549,206 | Intergenic | C/A | GWAS | 1400/5172 | 0.144/0.197 | 1.39x10^-7^ | 0.72 (0.64-0.81) | 3.89x10^-6^ | 0.151 | 0.000305 |
|  |  |  |  |  |  | Replication | 1960/4971 | 0.143/0.186 | 6.88x10^-7^ | 0.78 (0.70-0.87) | 5.81x10^-7^ | 0.00383 | 0.00176 |
|  |  |  |  |  |  | Combined | 3360/10143 | 0.144/0.192 | 2.32x10^-12^ | 0.75 (0.69-0.81) | 4.27x10^-12^ | 0.00305 | 1.38x10^-6^ |
| 14q21.1 | *---* | rs12887070 | 42,027,287 | Intergenic | A/C | GWAS | 1400/5172 | 0.070/0.048 | 2.84x10^-6^ | 1.52 (1.28-1.81) | 2.63x10^-6^ | 0.0438 | 0.00427 |
|  |  |  |  |  |  | Replication | 1960/4971 | 0.055/0.055 | 0.879 | 1.01 (0.85-1.20) | 0.126 | 0.00221 | 0.670 |
|  |  |  |  |  |  | Combined | 3360/10143 | 0.061/0.051 | 0.000801 | 1.23 (1.09-1.39) | 8.86x10^-6^ | 0.000251 | 0.0249 |
| 13q12.3 | *UBL3* | rs7994117 | 29,386,799 | Downstream | G/T | GWAS | 1400/5172 | 0.227/0.183 | 3.15x10^-6^ | 1.28 (1.16-1.42) | 3.34x10^-5^ | 0.157 | 8.39x10^-6^ |
|  |  |  |  |  |  | Replication | 1960/4971 | 0.202/0.205 | 0.520 | 0.97 (0.88-1.07) | 0.510 | 0.000893 | 0.589 |
|  |  |  |  |  |  | Combined | 3360/10143 | 0.212/0.193 | 0.00490 | 1.11 (1.03-1.19) | 0.0131 | 0.0474 | 0.000578 |
| 4p16.3 | *DGKQ* | rs11724804 | 955,779 | Intronic | A/G | GWAS | 1400/5172 | 0.485/0.436 | 6.83x10^-6^ | 1.22 (1.12-1.33) | 6.11x10^-5^ | 0.137 | 1.21x10^-5^ |
|  |  |  |  |  |  | Replication | 1960/4971 | 0.467/0.451 | 0.137 | 1.06 (0.98-1.15) | 0.00864 | 0.0147 | 0.703 |
|  |  |  |  |  |  | Combined | 3360/10143 | 0.575/0.443 | 1.99x10^-5^ | 1.13 (1.07-1.20) | 1.79x10^-6^ | 0.00477 | 0.000960 |
| 7p12.1 | *GRB10* | rs12540874 | 50,632,416 | Intronic | G/A | GWAS | 1400/5172 | 0.462/0.409 | 3.00x10^-6^ | 1.23 (1.13-1.34) | 0.000534 | 0.748 | 0.00169 |
|  |  |  |  |  |  | Replication | 1960/4971 | 0.416/0.395 | 0.0307 | 1.09 (1.01-1.18) | 0.0183 | 0.395 | 0.0935 |
|  |  |  |  |  |  | Combined | 3360/10143 | 0.435/0.403 | 1.27x10^-6^ | 1.15 (1.09-1.22) | 2.76x10^-5^ | 0.397 | 0.000647 |
| 2q37.1 | *AMRC9/PSMD1* | rs1868929 | 231,891,835 | Intronic | T/C | GWAS | 1400/5172 | 0.066/0.044 | 1.59x10^-6^ | 1.55 (1.30-1.86) | 3.20x10^-5^ | 0.387 | 0.0139 |
|  |  |  |  |  |  | Replication | 1960/4971 | 0.059/0.054 | 0.336 | 1.09 (0.92-1.28) | 0.0164 | 0.000651 | 0.974 |
|  |  |  |  |  |  | Combined | 3360/10143 | 0.062/0.048 | 7.67x10^-5^ | 1.28 (1.13-1.44) | 3.27x10^-6^ | 0.00137 | 0.112 |
