# Supplementary material for: Identification of Novel Genetic Markers Associated with Clinical Phenotypes of Systemic Sclerosis through a Genome-Wide Association Strategy
Source: PLoS Genet. 2011 Jul 14;7(7):e1002178. doi: 10.1371/journal.pgen.1002178 (PMC3136437; doi:10.1371/journal.pgen.1002178)
Supplement: Table S2 — Analysis for GWAS cohorts, replication cohorts and combined analysis for all non-HLA, non-previously described associations with dcSSc subtype of the disease. †P values for GWAS cohorts are Mantel-Haenszel meta-analysis GC corrected according to the set λ and in the replication and combined analysis Mantel-Haenszel meta-analysis P value. ‡P value for the totality of the SSc patients, in the case of GWAS cohorts GC corrected according to the set λ, and in replication and combined analysis Mantel-Haenszel meta-analysis P value. *Association in rs11171747 had a significant BD P value, thus making them heterogenic associations among populations. (DOC) [file pgen.1002178.s007.doc]

| Chr. | Gene | SNP | Base Pair | Location | Change | Stage | N (case/control) | MAF (case/control) | *P* value† | OR (CI 95%) | Full set *P*‡ | lcSSc *P*† | ATA+ *P*† |
| --- | --- | --- | --- | --- | --- | --- | --- | --- | --- | --- | --- | --- | --- |
| 7p12.1 | *AC009415.1* | rs2113648 | 52,029,517 | Intergenic | A/G | GWAS | 740/5172 | 0.277/0.225 | 6.21x10^-6^ | 1.33 (1.18-1.51) | 0.0128 | 0.958 | 0.00325 |
|  |  |  |  |  |  | Replication | 959/4971 | 0.229/0.246 | 0.164 | 0.92 (0.81-1.04) | 0.0660 | 0.109 | 0.661 |
|  |  |  |  |  |  | Combined | 1699/10143 | 0.251/0.234 | 0.0363 | 1.10 (1.01-1.20) | 0.651 | 0.266 | 0.0913 |
| 12q13.2 | *RPL41/ESYT1** | rs11171747 | 54,804,675 | Upstream | G/T | GWAS | 740/5172 | 0.446/0.385 | 2.19x10^-6^ | 1.31 (1.17-1.46) | 0.00176 | 0.433 | 0.00354 |
|  |  |  |  |  |  | Replication | 959/4971 | 0.408/0.372 | 0.00349 | 1.16 (1.05-1.29) | 0.000462 | 0.00379 | 0.0189 |
|  |  |  |  |  |  | Combined | 1699/10143 | 0.425/0.379 | 5.99x10^-8^ | 1.23 (1.14-1.33) | 1.76x10^-6^ | 0.00792 | 0.000174 |
| 13q33.2 | *EFNB2* | rs1477924 | 105,709,444 | Intergenic | G/A | GWAS | 740/5172 | 0.230/0.180 | 9.16x10^-6^ | 1.35 (1.19-1.54) | 0.000801 | 0.132 | 0.000800 |
|  |  |  |  |  |  | Replication | 959/4971 | 0.184/0.173 | 0.341 | 1.07 (0.93-1.22) | 0.312 | 0.395 | 0.269 |
|  |  |  |  |  |  | Combined | 1699/10143 | 0.205/0.177 | 0.000147 | 1.20 (1.09-1.31) | 0.00158 | 0.0911 | 0.00166 |
| 7q22.1 | *ZNF789* | rs10235235 | 98,913,767 | Intronic | C/T | GWAS | 740/5172 | 0.122/0.083 | 5.77x10^-6^ | 1.50 (1.26-1.78) | 0.0106 | 0.484 | 0.000168 |
|  |  |  |  |  |  | Replication | 959/4971 | 0.090/0.091 | 0.802 | 0.98 (0.82-1.17) | 0.111 | 0.0980 | 0.450 |
|  |  |  |  |  |  | Combined | 1699/10143 | 0.104/0.087 | 0.00337 | 1.20 (1.06-1.36) | 0.490 | 0.459 | 0.0457 |
| 7p15.1 | *JAZF1* | rs10275834 | 28,117,000 | Intronic | T/C | GWAS | 740/5172 | 0.314/0.263 | 5.28x10^-5^ | 1.28 (1.14-1.44) | 2.35x10^-5^ | 0.00803 | 0.0782 |
|  |  |  |  |  |  | Replication | 959/4971 | 0.270/0.279 | 0.551 | 0.97 (0.86-1.08) | 0.0520 | 0.0144 | 0.585 |
|  |  |  |  |  |  | Combined | 1699/10143 | 0.289/0.270 | 0.0193 | 1.10 (1.02-1.18) | 9.44x10^-6^ | 0.000244 | 0.108 |
