# Supplementary material for: Identification of Novel Genetic Markers Associated with Clinical Phenotypes of Systemic Sclerosis through a Genome-Wide Association Strategy
Source: PLoS Genet. 2011 Jul 14;7(7):e1002178. doi: 10.1371/journal.pgen.1002178 (PMC3136437; doi:10.1371/journal.pgen.1002178)
Supplement: Table S3 — Analysis for GWAS cohorts, replication cohorts and combined analysis for all non-HLA, non-previously described associations with ACA positive subgroup of the disease. †P values for GWAS cohorts are Mantel-Haenszel meta-analysis GC corrected according to the set λ and in the replication and combined analysis Mantel-Haenszel meta-analysis P value. ‡P value for the totality of the SSc patients, in the case of GWAS cohorts GC corrected according to the set λ, and in replication and combined analysis Mantel-Haenszel meta-analysis P value. *Association in rs3790567 had a significant BD P value, thus making them heterogeneous associations among populations. (DOC) [file pgen.1002178.s008.doc]

| Chr. | Gene | SNP | Base Pair | Location | Change | Stage | N (case/control) | MAF (case/control) | *P* value† | OR (CI 95%) | Full set *P*‡ | ATA+ *P*† | lcSSc *P*† |
| --- | --- | --- | --- | --- | --- | --- | --- | --- | --- | --- | --- | --- | --- |
| 1p13.3 | *IL12RB2* | rs3790567 | 67,594,965 | Intronic | A/G | GWAS | 761/5172 | 0.307/0.250 | 3.64x10^-6^ | 1.34 (1.18-1.51) | 2.97x10^-5^ | 0.0331 | 1.61x10^-5^ |
|  |  |  |  |  |  | Replication | 1030/4971 | 0.272/0.255 | 0.458 | 1.04 (0.93-1.17) | 0.00363 | 0.0663 | 0.0273 |
|  |  |  |  |  |  | Combined | 1791/10143 | 0.287/0.252 | 0.000197 | 1.17 (1.08-1.27) | 3.39x10^-7^ | 0.00468 | 3.51x10^-5^ |
| 21q22.12 | *RUNX1* | rs16993158 | 35,661,522 | Intronic | C/T | GWAS | 761/5172 | 0.107/0.080 | 6.48x10^-6^ | 1.53 (1.27-1.83) | 0.000334 | 0.330 | 0.000249 |
|  |  |  |  |  |  | Replication | 1030/4971 | 0.070/0.079 | 0.0916 | 0.85 (0.70-1.03) | 0.344 | 0.0745 | 0.524 |
|  |  |  |  |  |  | Combined | 1791/10143 | 0.086/0.077 | 0.0604 | 1.13 (0.99-1.29) | 0.00115 | 0.0444 | 0.00250 |
| 12p12.1 | *SOX5* | rs11047102 | 23,837,413 | Intronic | T/C | GWAS | 761/5172 | 0.133/0.097 | 1.03x10^-5^ | 1.46 (1.24-1.72) | 1.36x10^-6^ | 0.482 | 1.49x10^-7^ |
|  |  |  |  |  |  | Replication | 1030/4971 | 0.123/0.102 | 0.00291 | 1.27 (1.09-1.48) | 0.162 | 0.154 | 0.244 |
|  |  |  |  |  |  | Combined | 1791/10143 | 0.127/0.099 | 1.39x10^-7^ | 1.36 (1.21-1.52) | 7.52x10^-6^ | 0.121 | 5.11x10^-6^ |
