# Supplementary material for: Identification of Novel Genetic Markers Associated with Clinical Phenotypes of Systemic Sclerosis through a Genome-Wide Association Strategy
Source: PLoS Genet. 2011 Jul 14;7(7):e1002178. doi: 10.1371/journal.pgen.1002178 (PMC3136437; doi:10.1371/journal.pgen.1002178)
Supplement: Table S4 — Analysis for GWAS cohorts, replication cohorts and combined analysis for all non-HLA, non-previously described associations with ATA positive subgroup of the disease. †P values for GWAS cohorts are Mantel-Haenszel meta-analysis GC corrected according to the set λ and in the replication and combined analysis Mantel-Haenszel meta-analysis P value. ‡P value for the totality of the SSc patients, in the case of GWAS cohorts GC corrected according to the set λ, and in replication and combined analysis Mantel-Haenszel meta-analysis P value. (DOC) [file pgen.1002178.s009.doc]

| Chr. | Gene | SNP | Base Pair | Location | Change | Stage | N (case/control) | MAF (case/control) | *P* value† | OR (CI 95%) | Full set *P*‡ | ACA+ *P*† | dcSSc *P*† |
| --- | --- | --- | --- | --- | --- | --- | --- | --- | --- | --- | --- | --- | --- |
| 14q21.1 | *LRFN5* | rs1959429 | 40,973,264 | Intergenic | T/C | GWAS | 447/5172 | 0.172/0.245 | 8.97x10^-7^ | 0.63 (0.53-0.75) | 0.0189 | 0.106 | 0.0883 |
|  |  |  |  |  |  | Replication | 626/4971 | 0.277/0.249 | 0.0918 | 1.13 (0.98-1.30) | 0.00322 | 0.0353 | 0.0918 |
|  |  |  |  |  |  | Combined | 1073/10143 | 0.232/0.245 | 0.0371 | 0.89 (0.80-0.99) | 0.634 | 0.647 | 0.0371 |
| 15q14 | *ATPBD4/ZNF770* | rs4924647 | 33,282,199 | Intergenic | T/G | GWAS | 447/5172 | 0.086/0.050 | 2.76x10^-6^ | 1.85 (1.44-2.38) | 0.0130 | 0.746 | 0.00138 |
|  |  |  |  |  |  | Replication | 626/4971 | 0.062/0.061 | 0.938 | 1.01 (0.78-1.31) | 0.364 | 0.407 | 0.938 |
|  |  |  |  |  |  | Combined | 1073/10143 | 0.073/0.055 | 0.00172 | 1.33 (1.11-1.59) | 0.288 | 0.670 | 0.00172 |
| 4q35.1 | *DCTD/ODZ3* | rs4861533 | 184,028,292 | Intergenic | C/A | GWAS | 447/5172 | 0.301/0.231 | 1.50x10^-6^ | 1.46 (1.26-1.70) | 0.0319 | 0.535 | 0.00686 |
|  |  |  |  |  |  | Replication | 626/4971 | 0.243/0.238 | 0.746 | 1.02 (0.89-1.18) | 0.483 | 0.460 | 0.746 |
|  |  |  |  |  |  | Combined | 1073/10143 | 0.267/0.234 | 0.000424 | 1.20 (1.09-1.34) | 0.0412 | 0.884 | 0.000424 |
| 12p12.1 | *SOX5* | rs9634098 | 24,097,345 | Intergenic | T/C | GWAS | 447/5172 | 0.077/0.041 | 3.33x10^-6^ | 1.92 (1.46-2.51) | 0.00689 | 0.0133 | 0.0367 |
|  |  |  |  |  |  | Replication | 626/4971 | 0.043/0.045 | 0.247 | 0.84 (0.62-1.13) | 0.298 | 0.539 | 0.247 |
|  |  |  |  |  |  | Combined | 1073/10143 | 0.057/0.043 | 0.0265 | 1.25 (1.02-1.53) | 0.238 | 0.233 | 0.0265 |
