# Supplementary material for: Identification of Novel Genetic Markers Associated with Clinical Phenotypes of Systemic Sclerosis through a Genome-Wide Association Strategy
Source: PLoS Genet. 2011 Jul 14;7(7):e1002178. doi: 10.1371/journal.pgen.1002178 (PMC3136437; doi:10.1371/journal.pgen.1002178)
Supplement: Table S5 — Power calculations and genomic inflation factors (λ) in the whole SSc cohorts (GWAS and replication) and the lcSSc, dcSSc, ACA and ATA positive subphenotypes. 5×10^−8^ was used as significance threshold. (DOC) [file pgen.1002178.s010.doc]

| Phenotype | N | | λ | | | | | **OR 1.50** | | | | | | OR 1.30 | | | | | |
| --- | --- | --- | --- | --- | --- | --- | --- | --- | --- | --- | --- | --- | --- | --- | --- | --- | --- | --- | --- |
|  | Cases | Controls | All | US | Spain | Germany | Netherlands | MAF 0.40 | MAF 0.30 | **MAF 0.20** | MAF 0.15 | MAF 0.10 | MAF 0.05 | MAF 0.40 | MAF 0.30 | MAF 0.20 | MAF 0.15 | MAF 0.10 | MAF 0.05 |
| SSc | 5,471 | 10,143 | 1.081 | 1.090 | 1.033 | 1.126 | 1.045 | 100 | 100 | **100** | 100 | 100 | 100 | 100 | 100 | 100 | 100 | 94 | 34 |
| lcSSc | 3,360 | 10,143 | 1.058 | 1.071 | 1.033 | 1.101 | 1.028 | 100 | 100 | **100** | 100 | 100 | 90 | 100 | 100 | 99 | 93 | 64 | 10 |
| dcSSc | 1,699 | 10,143 | 1.034 | 1.049 | 1.032 | 1.061 | 1.020 | 100 | 100 | **100** | 100 | 93 | 29 | 94 | 88 | 64 | 39 | 12 | 1 |
| ACA+ | 1,791 | 10,143 | 1.050 | 1.051 | 1.042 | 1.081 | 1.012 | 100 | 100 | **100** | 100 | 95 | 33 | 96 | 91 | 69 | 43 | 14 | 1 |
| ATA+ | 1,073 | 10,143 | 1.061 | 1.035 | 1.025 | 1.078 | 1.030 | 100 | 100 | **98** | 89 | 53 | 5 | 62 | 47 | 22 | 9 | 2 | 0 |
