# Supplementary material for: Identification of Novel Genetic Markers Associated with Clinical Phenotypes of Systemic Sclerosis through a Genome-Wide Association Strategy
Source: PLoS Genet. 2011 Jul 14;7(7):e1002178. doi: 10.1371/journal.pgen.1002178 (PMC3136437; doi:10.1371/journal.pgen.1002178)
Supplement: Table S6 — Conditional logistic regression analysis of all the independently associated SNPs in the HLA region in the ACA positive patients. †P values for Mantel-Haenszel meta-analysis GC corrected according to the set λ. (DOC) [file pgen.1002178.s011.doc]

|  |  |  |  | Conditioned to rs9275390 | | Conditioned to rs6457617 | | Conditioned to rs443198 | |
| --- | --- | --- | --- | --- | --- | --- | --- | --- | --- |
| SNP | BP | *P* Value† | OR | *P* value | OR | *P* value | OR | *P* value | OR |
| rs9275390 | 32,777,134 | 2.62x10^-54^ | 2.385 | NA | NA | 1.52x10^-67^ | 1.972 | 1.14x10^-130^ | 2.267 |
| rs6457617 | 32,771,829 | 1.99x10^-36^ | 0.477 | 5.14x10^-67^ | 0.703 | NA | NA | 6.83x10^-105^ | 0.513 |
| rs443198 | 32,298,384 | 8.84x10^-21^ | 0.556 | 8.11x10^-12^ | 0.691 | 7.43x10^-11^ | 0.662 | NA | NA |
