# Supplementary material for: Identification of Novel Genetic Markers Associated with Clinical Phenotypes of Systemic Sclerosis through a Genome-Wide Association Strategy
Source: PLoS Genet. 2011 Jul 14;7(7):e1002178. doi: 10.1371/journal.pgen.1002178 (PMC3136437; doi:10.1371/journal.pgen.1002178)
Supplement: Table S7 — Conditional logistic regression analysis of all the independently associated SNPs in the HLA region in the ATA positive patients. †P values for Mantel-Haenszel meta-analysis GC corrected according to the set λ. (DOC) [file pgen.1002178.s012.doc]

|  |  |  |  | Conditioned to rs3129882 | | Conditioned to rs987870 | | Conditioned to rs3135021 | | Conditioned to rs3129763 | | Conditioned to rs9296015 | | Conditioned to rs1810472 | | Conditioned to rs6901221 | |
| --- | --- | --- | --- | --- | --- | --- | --- | --- | --- | --- | --- | --- | --- | --- | --- | --- | --- |
| SNP | BP | *P* Value† | OR | *P* value | OR | *P* value | OR | *P* value | OR | *P* value | OR | *P* value | OR | *P* value | OR | *P* value | OR |
| rs3129882 | 32,517,508 | 1.89x10^-27^ | 2.168 | NA | NA | 3.89x10^-26^ | 2.359 | 2.95x10^-27^ | 2.097 | 2.39x10^-23^ | 2.014 | 2.23x10^-29^ | 2.038 | 1.19x10^-36^ | 2.170 | 1.69x10^-30^ | 2.166 |
| rs987870 | 33,150,858 | 2.41x10^-20^ | 2.093 | 6.01x10^-16^ | 2.564 | NA | NA | 7.43x10^-25^ | 2.501 | 1.09x10^-15^ | 2.144 | 3.55x10^-15^ | 2.295 | 7.73x10^-10^ | 1.988 | 8.51x10^-19^ | 2.274 |
| rs3135021 | 33,153,536 | 1.95x10^-12^ | 1.656 | 1.47x10^-60^ | 1.658 | 3.18x10^-121^ | 1.935 | NA | NA | 2.50x10^-57^ | 1.657 | 2.33x10^-77^ | 1.745 | 6.07x10^-55^ | 1.568 | 2.78x10^-88^ | 1.783 |
| rs3129763 | 32,698,903 | 1.47x10^-11^ | 1.647 | 9.42x10^-149^ | 1.442 | 3.04x10^-176^ | 1.596 | 4.47x10^-179^ | 1.604 | NA | NA | 0 | 1.574 | 9.19x10^-187^ | 1.723 | 7.61x10^-112^ | 1.726 |
| rs9296015 | 32,326,967 | 1.14x10^-8^ | 0.545 | 2.07x10^-7^ | 0.706 | 1.17x10^-14^ | 0.546 | 1.49x10^-18^ | 0.561 | 9.98x10^-11^ | 0.633 | NA | NA | 1.27x10^-16^ | 0.566 | 2.63x10^-13^ | 0.594 |
| rs1810472 | 33,191,099 | 2.14x10^-8^ | 1.498 | 1.38x10^-36^ | 1.583 | 2.21x10^-8^ | 1.251 | 7.33x10^-26^ | 1.363 | 8.48x10^-45^ | 1.604 | 3.31x10^-36^ | 1.570 | NA | NA | 4.96x10^-39^ | 1.513 |
| rs6901221 | 33,206,254 | 2.54x10^-8^ | 1.606 | 1.51x10^-9^ | 1.612 | 9.73x10^-23^ | 1.613 | 2.23x10^-13^ | 1.659 | 7.49x10^-16^ | 1.643 | 1.23x10^-9^ | 1.516 | 9.47x10^-12^ | 1.504 | NA | NA |
