# Supplementary material for: Identification of Novel Genetic Markers Associated with Clinical Phenotypes of Systemic Sclerosis through a Genome-Wide Association Strategy
Source: PLoS Genet. 2011 Jul 14;7(7):e1002178. doi: 10.1371/journal.pgen.1002178 (PMC3136437; doi:10.1371/journal.pgen.1002178)
Supplement: Table S8 — Independent associations found in the HLA region in the ACA positive subgroup of patients in the separate four GWAS cohorts. †Uncorrected χ2 P value of each separated cohort. (DOC) [file pgen.1002178.s013.doc]

| SNP | Gene | Location | Change | Population | MAF (case/control) | *P*† | OR (CI 95%) |
| --- | --- | --- | --- | --- | --- | --- | --- |
| rs443198 | *NOTCH4* | Exon | C/T | Spain | 0.295/0.414 | 0.000190 | 0.593 (0.45-0.78) |
|  |  |  |  | Germany | 0.240/0.339 | 0.00236 | 0.615 (0.45-0.84) |
|  |  |  |  | Netherlands | 0.233/0.327 | 0.0692 | 0.624 (0.37-1.04) |
|  |  |  |  | US | 0.243/0.380 | 3.40x10^-15^ | 0.524 (0.45-0.62) |
| rs6457617 | *HLA-DQB1* | Intergenic | C/T | Spain | 0.325/0.474 | 4.81x10^-6^ | 0.535 (0.41-0.70) |
|  |  |  |  | Germany | 0.298/0.504 | 3.40x10^-9^ | 0.417 (0.31-0.56) |
|  |  |  |  | Netherlands | 0.430/0.511 | 0.147 | 0.723 (0.47-1.12) |
|  |  |  |  | US | 0.302/0.488 | 9.38x10^-25^ | 0.455 (0.39-0.53) |
| rs9275390 | *HLA-DQB1* | Intergenic | C/T | Spain | 0.458/0.306 | 1.20x10^-6^ | 1.920 (1.47-2.50) |
|  |  |  |  | Germany | 0.475/0.250 | 6.45x10^-13^ | 2.733 (2.06-3.62) |
|  |  |  |  | Netherlands | 0.361/0.269 | 0.0664 | 1.530 (0.97.2.42) |
|  |  |  |  | US | 0.456/0.245 | 1.09x10^-39^ | 2.585 (2.24-2.99) |
