# Supplementary material for: Identification of Novel Genetic Markers Associated with Clinical Phenotypes of Systemic Sclerosis through a Genome-Wide Association Strategy
Source: PLoS Genet. 2011 Jul 14;7(7):e1002178. doi: 10.1371/journal.pgen.1002178 (PMC3136437; doi:10.1371/journal.pgen.1002178)
Supplement: Table S9 — Independent associations found in the HLA region in the ATA positive subgroup of patients in the separate four GWAS cohorts. †Uncorrected χ2 P value of each separated cohort. (DOC) [file pgen.1002178.s014.doc]

| SNP | Gene | Location | Change | Population | MAF (case/control) | *P*† | OR (CI 95%) |
| --- | --- | --- | --- | --- | --- | --- | --- |
| rs9296015 | *NOTCH4* | Intergenic | A/G | Spain | 0.161/0.280 | 0.001614 | 0.492 (0.31-0.77) |
|  |  |  |  | Germany | 0.077/0.174 | 0.00145 | 0.399 (0.22-0.72) |
|  |  |  |  | Netherlands | 0.148/0.187 | 0.3581 | 0.754 (0.42-1.38) |
|  |  |  |  | US | 0.111/0.178 | 0.000217 | 0.580 (0.43-0.78) |
| rs3129882 | *HLA-DRA* | Intron | G/A | Spain | 0.593/0.448 | 0.000804 | 1.793 (1.27.2.53) |
|  |  |  |  | Germany | 0.691/0.444 | 1.64x10^-9^ | 2.792 (1.98-3.94) |
|  |  |  |  | Netherlands | 0.580/0.388 | 0.000393 | 2.173 (1.40-3.37) |
|  |  |  |  | US | 0.632/0.448 | 6.35x10^-15^ | 2.117 (1.75-2.57) |
| rs3129763 | *HLA-DQA1/DRB1* | Intergenic | A/G | Spain | 0.340/0.231 | 0.00356 | 1.716 (1.19-2.48) |
|  |  |  |  | Germany | 0.363/0.245 | 0.000952 | 1.759 (1.25-2.47) |
|  |  |  |  | Netherlands | 0.330/0.239 | 0.0550 | 1.568 (0.99-2.49) |
|  |  |  |  | US | 0.349/0.250 | 1.81x10^-6^ | 1.606 (1.32-1.96) |
| rs987870 | *HLA-DPA1/DPB1* | Intron | C/T | Spain | 0.333/0.191 | 6.66x10^-5^ | 2.112 (1.46-3.08) |
|  |  |  |  | Germany | 0.310/0.133 | 1.92x10^-9^ | 2.926 (2.04-4.21) |
|  |  |  |  | Netherlands | 0.250/0.156 | 0.0206 | 1.804 (1.09-2.99) |
|  |  |  |  | US | 0.240/0.142 | 6.25x10^-9^ | 1.907 (1.53-2.38) |
| rs3135021 | *HLA-DPA1/DPB1* | Intron | A/G | Spain | 0.438/0.328 | 0.00722 | 1.601 (1.13-2.26) |
|  |  |  |  | Germany | 0.369/0.271 | 0.00769 | 1.574 (1.13-2.20) |
|  |  |  |  | Netherlands | 0.363/0.280 | 0.0934 | 1.468 (0.94-2.31) |
|  |  |  |  | US | 0.410/0.285 | 8.29x10^-9^ | 1.738 (1.44-2.10) |
| rs6901221 | *HLA-DPA1/DPB1* | Intron | C/A | Spain | 0.160/0.103 | 0.0352 | 1.667 (1.03-2.69) |
|  |  |  |  | Germany | 0.244/0.172 | 0.0212 | 1.558 (1.07-2.28) |
|  |  |  |  | Netherlands | 0.227/0.180 | 0.267 | 1.340 (0.80-2.25) |
|  |  |  |  | US | 0.235/0.156 | 4.85x10^-6^ | 1.668 (1.38-2.08) |
