# Supplementary material for: Identification of Novel Genetic Markers Associated with Clinical Phenotypes of Systemic Sclerosis through a Genome-Wide Association Strategy
Source: PLoS Genet. 2011 Jul 14;7(7):e1002178. doi: 10.1371/journal.pgen.1002178 (PMC3136437; doi:10.1371/journal.pgen.1002178)
Supplement: Table S10 — Previously described genetic associations with SSc subphenotypes which were present in the present study's GWAS panel of SNPs. A total of 2,296 SSc cases and 5,172 controls were included in this analysis. The SSc cases included 1,400 lcSSc individuals, 740 dcSSc individuals, 761 ACA+ individuals and 447 ATA+ individuals. Best P value in each subgroup for each SNP is in bold. Chr. Chromosome. † Uncorrected Mantel-Haenszel Meta-analysis P value of the four GWAS cohorts. (DOC) [file pgen.1002178.s015.doc]

| Chr. | Gene | SNP | Base Pair | Change | lcSSc | | dcSSc | | ACA+ | | ATA+ | | Refs |
| --- | --- | --- | --- | --- | --- | --- | --- | --- | --- | --- | --- | --- | --- |
|  |  |  |  |  | *P*^†^ value | OR (95% CI) | *P*^†^ value | OR (95% CI) | *P*^†^ value | OR (95% CI) | *P*^†^ value | OR (95% CI) |  |
| 1 | *CD247* | rs2056626 | 165,687,049 | G/T | 2.66x10^-6^ | 0.81 (0.75-0.89) | 7.70x10^-3^ | 0.85 (0.78-0.96) | 7.10x10^-3^ | 0.86 (0.77-0.96) | 3.22x10^-5^ | 0.74 (0.64-0.85) | [12] |
| 1 | *TNFSF4* | rs2205960 | 171,458,098 | T/G | 7.70x10^-4^ | 1.18 (1.03-1.31) | 0.506 | 1.05 (0.92-1.19) | 0.0162 | 1.17 (1.03-1.33) | 9.05x10^-3^ | 1.23 (1.05-1.45) | [13,14] |
| 2 | *STAT4* | rs3821236 | 191,611,003 | A/G | 8.86x10^-8^ | 1.31 (1.19-1.48) | 5.96x10^-4^ | 1.25 (1.10-1.43) | 1.18x10^-4^ | 1.29 (1.47-4.37) | 1.53x10^-3^ | 1.30 (1.11-1.52) | [4, 6, 7, 8] |
| 4 | *BANK1* | rs10516487 | 102,970,099 | T/C | 0.317 | 1.05 (0.96-1.15) | 0.0103 | 0.85 (0.75-0.96) | 0.140 | 1.09 (0.97-1.23) | 0.109 | 0.88 (0.75-1.03) | [5, 9] |
| 7 | *IRF5* | rs10488631 | 128,381,419 | C/T | 1.64x10^-10^ | 1.50 (1.32-1.69) | 1.27x10^-9^ | 1.61 (1.38-1.88) | 1.88x10^-7^ | 1.52 (1.30-179) | 8.25x10^-7^ | 1.63 (1.34-1.98) | [15] |
| 8 | *BLK* | rs2736340 | 11,381,382 | T/C | 1.54x10^-4^ | 1.20 (1.09-1.32) | 1.39x10^-3^ | 1.22 (1.08-1.38) | 1.45x10^-4^ | 1.27 (1.12-1.44) | 0.387 | 1.07 (0.92-1.26) | [10, 11] |
