# Supplementary material for: Identification of Novel Genetic Markers Associated with Clinical Phenotypes of Systemic Sclerosis through a Genome-Wide Association Strategy
Source: PLoS Genet. 2011 Jul 14;7(7):e1002178. doi: 10.1371/journal.pgen.1002178 (PMC3136437; doi:10.1371/journal.pgen.1002178)
Supplement: Table S11 — Composition and size of all the populations used in the study for the considered features of the disease. (DOC) [file pgen.1002178.s016.doc]

| Population | Population Size | | Sex (cases/controls) | | Subtype | | ACA Positive | ATA Positive |
| --- | --- | --- | --- | --- | --- | --- | --- | --- |
|  | Cases | Controls | Female | Male | Diffuse | Limited |  |  |
| Overall | 5471 | 10143 | 0.86/0.79 | 0.14/0.21 | 0.34 | 0.66 | 0.37 | 0.22 |
| **GWAS SSc cohorts** |  |  |  |  |  |  |  |  |
| Spain | 364 | 384 | 0.88/0.75 | 0.10/0.25 | 0.30 | 0.64 | 0.42 | 0.20 |
| Germany | 270 | 671 | 0.88/0.62 | 0.11/0.38 | 0.40 | 0.55 | 0.42 | 0.31 |
| The Netherlands | 176 | 639 | 0.72/0.51 | 0.28/0.49 | 0.28 | 0.51 | 0.22 | 0.26 |
| US | 1486 | 3478 | 0.88/0.88 | 0.12/0.12 | 0.36 | 0.64 | 0.32 | 0.17 |
| **Replication SSc cohorts** | |  |  |  |  |  |  |  |
| US | 616 | 1143 | 0.88/0.44 | 0.12/0.56 | 0.41 | 0.59 | 0.32 | 0.15 |
| Belgium | 187 | 272 | 0.78/0.45 | 0.22/0.54 | 0.33 | 0.67 | 0.33 | 0.25 |
| Italy | 500 | 509 | 0.92/0.65 | 0.08/0.35 | 0.26 | 0.74 | 0.42 | 0.33 |
| Sweden | 268 | 278 | 0.80/0.78 | 0.20/0.22 | 0.27 | 0.73 | 0.28 | 0.18 |
| UK | 485 | 380 | 0.84/0.44 | 0.16/0.56 | 0.27 | 0.73 | 0.38 | 0.15 |
| Norway | 113 | 282 | 0.85/--- | 0.15/--- | 0.35 | 0.65 | 0.54 | 0.14 |
| Spain | 626 | 705 | 0.87/0.60 | 0.11/0.37 | 0.32 | 0.68 | 0.47 | 0.23 |
| The Netherlands | 204 | 350 | 0.71/0.45 | 0.29/0.54 | 0.32 | 0.68 | 0.25 | 0.27 |
| Germany | 176 | 291 | 0.81/0.44 | 0.13/0.27 | 0.42 | 0.58 | 0.39 | 0.32 |
