# Supplementary material for: Identification of Novel Genetic Markers Associated with Clinical Phenotypes of Systemic Sclerosis through a Genome-Wide Association Strategy
Source: PLoS Genet. 2011 Jul 14;7(7):e1002178. doi: 10.1371/journal.pgen.1002178 (PMC3136437; doi:10.1371/journal.pgen.1002178)
Supplement: Text S1 — Members of the Spanish Scleroderma Group. (DOC) [file pgen.1002178.s017.doc]

**Supplementary Note**

**Spanish Scleroderma Group**

**Rosa García Portales**, Hospital Virgen de la Victoria, Málaga; **Mayte Camps** and **Antonio Fernández-Nebro**, Hospital Carlos Haya, Málaga; **Jose Román-Ivorra** and **Emma Beltran**, Hospital Dr. Peset, Valencia; **Luis Rodríguez-Rodríguez**, Hospital Clínico San Carlos, Madrid; **Mª Jesús Castillo** and **Julio Sánchez-Román**, Hospital Virgen del Rocío, Sevilla; **Esther Vicente**, Hospital La Princesa, Madrid; **Bernardino Díaz**, **Luis Trapiella** and **María Gallego**, Hospital Central de Asturias, Oviedo; **Mª Ángeles Aguirre**, Hospital Reina Sofía, Córdoba; **Jose Luis Callejas-Rubio** and **Raquel Ríos**, Hospital San Cecilio, Granada; **Iván Castellvi**, Hospital Sant Pau, Barcelona; **Mª Victoria Egurbide**, Hospital de Cruces, Vizcaya; **Gerard Espinosa**, Hospital Clinic, Barcelona; **Anna Pros**, Hospital del Mar, Barcelona; **Nuria Navarrete**, Hospital Virgen de las Nieves, Granada; **Federico Díaz-González**, Hospital Universitario de Canarias, Tenerife; **José Luis Andréu** and **Mónica Fernández-Castro**, Hospital Puerta del Hierro, Madrid; **Luis Sáez Comet**, Hospital Miguel Servet, Zaragoza; **Francisco J. López-Longo**, Hospital Gregorio Marañón, Madrid.
