# Supplementary material for: Identification of Novel Genetic Markers Associated with Clinical Phenotypes of Systemic Sclerosis through a Genome-Wide Association Strategy
Source: PLoS Genet. 2011 Jul 14;7(7):e1002178. doi: 10.1371/journal.pgen.1002178 (PMC3136437; doi:10.1371/journal.pgen.1002178)
Supplement: Text S2 — URLs. Internet Uniform Resource Locator (URL) for each of the software packages used in this study. (DOC) [file pgen.1002178.s018.doc]

**URLs**

Plink software:

<http://pngu.mgh.harvard.edu/purcell/plink/>

SVS HelixTree software:

<http://www.goldenhelix.com/SNP_Variation/HelixTree/index.html>

Stata software:

<http://www.stata.com/>

R Statistical Package:

<http://www.r-project.org/>

Haploview:

<http://www.broadinstitute.org/scientific-community/science/programs/medical-and-population-genetics/haploview/haploview>
